# Ten-eleven translocation protein 1 modulates medulloblastoma progression

**DOI:** 10.1186/s13059-021-02352-9

**Published:** 2021-04-29

**Authors:** Hyerim Kim, Yunhee Kang, Yujing Li, Li Chen, Li Lin, Nicholas D. Johnson, Dan Zhu, M. Hope Robinson, Leon McSwain, Benjamin G. Barwick, Xianrui Yuan, Xinbin Liao, Jie Zhao, Zhiping Zhang, Qiang Shu, Jianjun Chen, Emily G. Allen, Anna M. Kenney, Robert C. Castellino, Erwin G. Van Meir, Karen N. Conneely, Paula M. Vertino, Peng Jin, Jian Li

**Affiliations:** 1 Department of Human Genetics, Emory University School of Medicine, Atlanta, GA 30322 USA; 2 Department of Biostatistics and Health Data Science, Indiana University School of Medicine, Indianapolis, IN 46202 USA; 3 Laboratory of Molecular Neuro-Oncology, Department of Neurosurgery, Emory University School of Medicine, Atlanta, GA 30322 USA; 4 Department of Hematology and Medical Oncology, Emory University School of Medicine, Atlanta, GA 30322 USA; 5 Department of Pediatric Oncology, Emory University School of Medicine, Atlanta, GA 30322 USA; 6 Winship Cancer Institute, Emory University, Atlanta, GA 30322 USA; 7 Department of Neurosurgery, Xiangya Hospital, Central South University, Changsha, 410008 Hunan China; 8 Hydrocephalus Center, Xiangya Hospital, Central South University, Changsha, 410008 Hunan China; 9 The Children’s Hospital and Institute of Translational Medicine, School of Medicine, Zhejiang University, Hangzhou, China; 10 Department of Systems Biology and Gehr Family Center for Leukemia Research, City of Hope, Duarte, CA 91010 USA; 11 Aflac Cancer and Blood Disorders Center, Children’s Healthcare of Atlanta, Emory University School of Medicine, Atlanta, GA 30322 USA

**Keywords:** Medulloblastoma, 5-hydroxymethylcytosine, TET1, Stem-like property, NANOG, PDGF signaling pathway

## Abstract

**Background:**

Medulloblastoma (MB) is the most common malignant pediatric brain tumor that originates in the cerebellum and brainstem. Frequent somatic mutations and deregulated expression of epigenetic regulators in MB highlight the substantial role of epigenetic alterations. 5-hydroxymethylcytosine (5hmC) is a highly abundant cytosine modification in the developing cerebellum and is regulated by ten-eleven translocation (TET) enzymes.

**Results:**

We investigate the alterations of 5hmC and TET enzymes in MB and their significance to cerebellar cancer formation. We show total abundance of 5hmC is reduced in MB, but identify significant enrichment of MB-specific 5hmC marks at regulatory regions of genes implicated in stem-like properties and Nanog-binding motifs. While TET1 and TET2 levels are high in MBs, only knockout of *Tet1* in the smoothened (*SmoA1)* mouse model attenuates uncontrolled proliferation, leading to a favorable prognosis. The pharmacological *Tet1* inhibition reduces cell viability and *platelet-derived growth factor* signaling pathway-associated genes.

**Conclusions:**

These results together suggest a potential key role of 5hmC and indicate an oncogenic nature for TET1 in MB tumorigenesis, suggesting it as a potential therapeutic target for MBs.

## Background

Medulloblastoma (MB) is the most common malignant pediatric brain tumor that originates in the cerebellum and brainstem. While this embryonal tumor has a lower mutation rate than adult solid tumors, frequent somatic mutations and deregulated expression of epigenetic regulators, including chromatin remodeling genes and histone modifiers, highlight the substantial role of epigenetic alterations in its formation/development/genesis [1, 2]. Indeed, DNA methylation signature is a key factor for MB molecular stratification, together with transcriptional signature, into four groups: Wingless (WNT), Sonic Hedgehog (SHH), Group 3, and Group 4 [2–5]. Studies in mice also support the importance of epigenome regulation in MB: targeting chromatin remodeling with a combination of DNA methyltransferases (DNMT) inhibitor 5-aza-2′deoxycytidine (5-aza-dC) and histone deacetylases (HDAC) inhibitor valproic acid (VPA) effectively inhibits tumor formation in the patched homolog (*Ptch)* heterozygous mice, a model of SHH-MB [6], and antagonizing methyl-CpG binding protein methyl-CpG binding domain protein 2 (MBD2) inhibits MB xenografts [7]. Despite the significance of the epigenome in MB, there have been limited studies investigating the dynamic nature of cytosine modifications and enzymes involved in the process.

In humans, cerebellar development involves active neuronal maturation and circuit formation from the third trimester up to 2 years after birth [8, 9]; thus, precise and timely gene regulation during this period is critical for neurogenesis from neural progenitors. In mice, this process is similar, but is easier to study as it occurs within the first 3 weeks postnatally. During murine cerebellar maturation, while a small increase in global levels of 5-methylcytosine (5mC) is detected, a much more dramatic change in the abundance of its oxidative derivative, 5-hydroxymethylcytosine (5hmC), occurs, reaching approximately 0.4 to 0.9% of total cytosines [10, 11]. In addition, 5hmC is enriched at cerebellar-specific enhancers and the exon start site of highly expressed genes involved in axon guidance and ion channels [11, 12]. This evidence suggests 5hmC plays a role in establishing and maintaining cell identity during the period of circuit formation. Interestingly, the abundance of 5hmC is significantly reduced in many solid tumors, such as melanoma [13], prostate, breast, liver, colon cancers [14, 15], and brain tumors [15–17], which is associated with shorter postoperative survival [18]. Moreover, enzymatic impairment caused by somatic mutations and copy number alterations or the deregulated expression of TET enzymes is frequently identified in many cancers and is often associated with unfavorable prognosis [19–22].

To investigate the alterations of 5hmC and TET enzymes in MB and their significance to cerebellar cancer formation, we employed genome-wide 5hmC profiling and the *SmoA1* mouse MB model. Consistent with previous findings in different tumors, we show that 5hmC is depleted in MB, but identify significant enrichment of MB-specific 5hmC marks at regulatory regions of genes implicated in stem-like properties and Nanog-binding motifs. Moreover, the abolishment of *Tet1* yielded a more favorable prognosis in the *SmoA1* mouse MB model, and pharmacological inhibition of *Tet1* expression reduced cell viability and suppressed *platelet-derived growth factor (*PDGF) signaling pathway. Collectively, these data suggest that upregulated *Tet1* may contribute to maintaining stem-like properties in MB, validating it as a potential therapeutic target for MBs.

## Results

### Decreased 5hmC level is associated with MB prognosis

**Fig. 1:**
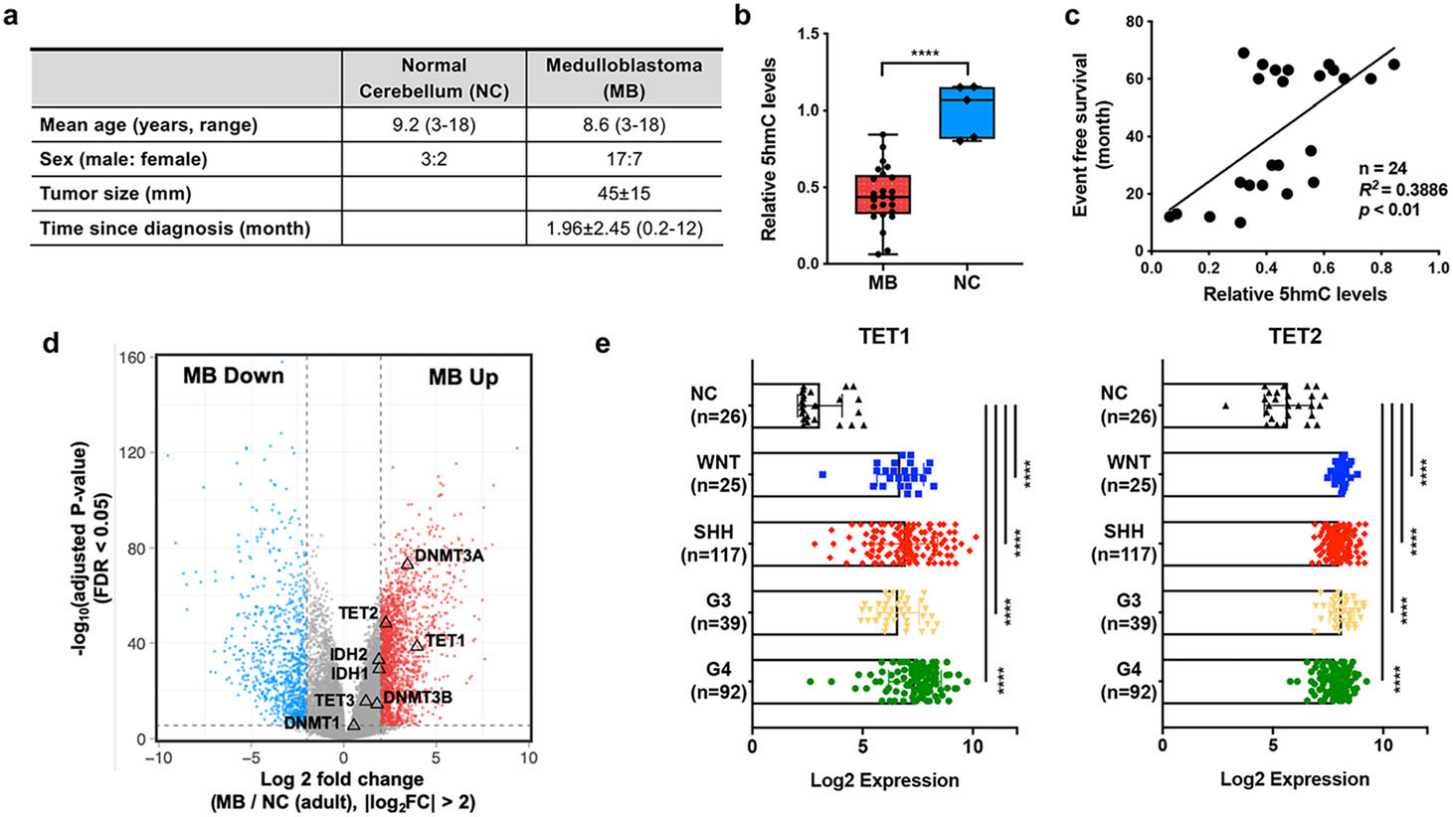
Loss of 5-hydroxymethylation is a hallmark of MBs. **a** Table illustrates sample information (age, sex, tumor size, and time since diagnosis) of MBs and normal cerebella (NC). **b** UHPLC-MS/MS analysis shows a significant decrease of total 5hmC levels in MBs (*n* = 24) compared with age-matched normal cerebella (NC) (*n* = 5) (****p < 0.001). **c** Linear correlation between 5hmC abundance in MBs and prognosis. Lower 5hmC level is associated with worse prognosis (*R*^*2*^ = 0.3866, *p* < 0.01). **d** Volcano plot showing distribution of differentially expressed genes in MBs, with log2 fold change of MB/NC on *x*-axis and *P* values on *y*-axis. Genes with log2 fold change > 2 are indicated in red, and genes with log2 fold change < − 2 are indicated in blue (FDR < 0.05). **e** Expression level of TET1 and TET2 in each molecular subgroup of MB (*n* = 273) and NC (*n* = 26) (*p* < 0.001). G3: group3, G4: group 4

Previous studies have found significant depletion of 5hmC across many human cancer genomes compared to those of corresponding normal tissues [13, 23–26]. To further explore this epigenetic characteristic in MB, we first examined 5hmC levels using ultra-high-performance liquid chromatography–tandem mass-spectrometry (UHPLC-MS/MS) analysis in normal cerebellar tissues without neurological disorders (*n* = 5) and in primary MB tissues (*n* = 24). In normal tissues from children between 3 and 18 years of age, 5hmC levels were confined to a narrow range (0.80- to 1.15-fold compared to the mean value (Fig. 1a, b) [12]. In contrast, we identified substantially lower levels in MB tissues with an average of 0.44-fold global 5hmC levels relative to normal of comparable age (*p* < 0.001; Fig. 1a, b). In an independent cohort, we consistently observed a significant reduction of 5hmC levels in MB (*n* = 5) compared to age-matched normal without marked neurological disorders (*n* = 6) using both dot blot assay and UHPLC-MS/MS analysis (Additional file 1: Fig. S1a, b), suggesting that the reduction of 5hmC is a characteristic for MB. Although there was little variation in 5hmC levels between normal cerebellar tissues, MB exhibited significant intertumoral variation in 5hmC levels with 0.06 to 0.84-fold differences (Fig. 1a, b). In some cases, 5hmC levels were almost undetected whereas for other levels were similar to those seen in normal tissues (Fig. 1b). A notable linear correlation between 5hmC levels and prognosis (*n* = 24, *R*^2^ = 0.3886, *p* < 0.001; Fig. 1c) was also observed and was a better indicator of prognosis than age at diagnosis (Additional file 1: Fig. S1c).

### Dysregulation of TET expression in MBs

We next investigated the cause of 5hmC depletion in MB. DNA methylation is a prerequisite for 5hmC generation in vivo; therefore, genomic hypomethylation can lead to loss of 5hmC in tumors. In addition, 5hmC loss results from hyperproliferation, as there is no mechanism of maintaining 5hmC levels [27]. Other plausible mechanisms are inactivating mutations of *TET* enzymes (*TET1, TET2, TET3*) which are responsible for converting 5mC to 5hmC or indirect inhibition of TET enzymatic activity caused by the oncometabolite 2-hydroxyglutarate that accumulates in tumors with isocitrate dehydrogenase 1/2 (*IDH1/2)* mutations and interferes with α-ketoglutarate-dependent oxygenases, including the TET enzymes [28, 29]. Querying four publicly available datasets (from Broad, the International Cancer Genome Consortium (ICGC), the Pediatric Cancer Genome Project (PCGP) and SickKids using cBioPortal [30, 31]) showed only three out of 300 patients had putative driving mutations in any of the TET enzymes (truncating mutations of *TET1* or *TET2*) or missense mutation *of IDH1* genes, indicating that genetic alterations are not the cause of loss of 5hmC in most cases (Additional file 1: Fig. S1d). In addition, no focal amplification/deletion of enzymes involved in cytosine dynamics has been observed in previous studies [32]. To explore deregulated expression patterns of enzymes involved in 5hmC generation, we performed a meta-analysis of 8 different publicly available gene expression datasets containing 273 human MB samples and 31 fetal and adult human cerebellar tissue controls (Additional file 2: Table S1). This analysis revealed significant upregulation of *DNMT3A*, *TET1*, and *TET2* genes in MB compared to adult normal controls (FDR < 0.05 and log2 fold changes > 2, Fig. 1d), at expression levels comparable to those observed in fetal cerebellar tissues (Additional file 1: Fig. S1). Since TET enzymes are more directly involved in 5hmC formation, we further investigated whether *TET1* and *TET2* expression is regulated by particular pathways within the different MB groups. While we observed intertumoral variation, there is no significant difference in *TET1/2* mRNA levels across MB molecular groups (Fig. 1e), suggesting that their regulation may be independent of the distinct signaling pathways found in each subgroup. Given that high levels of *TET1* and *TET2* mediate epigenetic reprogramming by passive and active DNA demethylation processes in primordial germ cells (PGCs) [33–35], high levels of TET1/2 in MB could underlie global hypomethylation in PGC [36].

### Distinct 5hmC signature in MBs

**Fig. 2:**
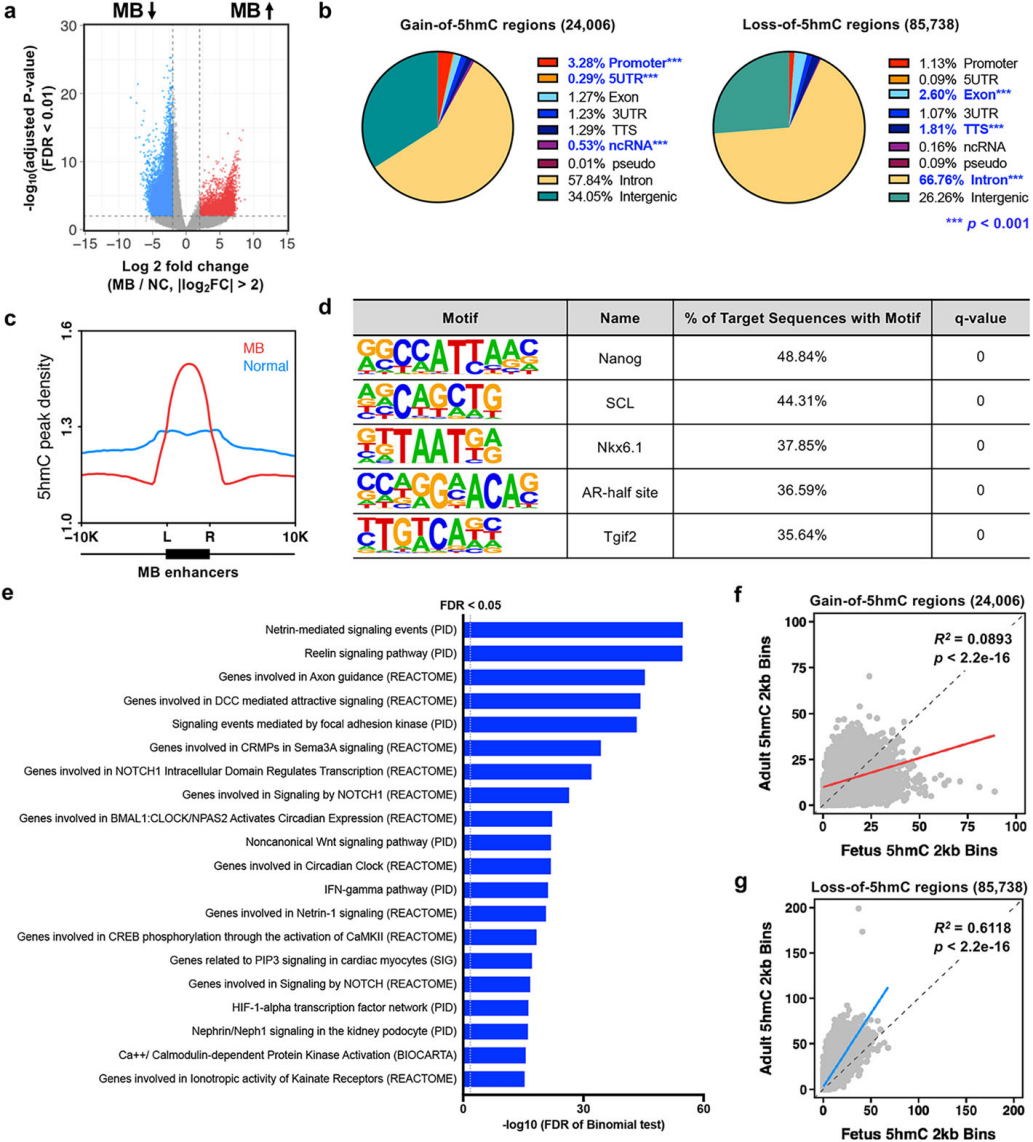
MB-associated DhMRs are implicated in stem-like properties. **a** Volcano plot showing the hMe-seal data of 16 MB and 6 NC samples. Red dots indicate gain-of-5hmC regions in MB, and blue dots indicate loss-of-5hmC regions in MB. Two-thousand-base-pair binning was performed, and the criteria were set as an absolute value of the log2 fold change (MB/NC) > 2 and FDR < 0.01. **b** Genomic annotation of identified 5hmC gain or 5hmC loss in MBs using HOMER. Annotations with FDR < 0.001 compared to background are highlighted in blue. **c** Plot of average 5hmC containing 2 kb bins around previously reported MB active enhancers. **d** Sequence logos for the highly enriched sequence motifs in 5hmC acquiring genomic regions in MBs. **e** Top 20 pathways identified by Molecular Signatures Database (MSigDB) enrichment analysis with GREAT using 5hmC gain in MB. Each term shows statistical significance in both binomial test and hypergeometric test (FDR < 0.05). **f**, **g** Plots using mapped 5hmC containing bins of fetus and adult samples at either 5hmC gain in MBs (*n* = 24,006, top) or 5hmC loss in MBs (*n* = 85,738, bottom). Sequencing reads of fetus and adult are used to generate binned matrices (binsize: 2 kb). Linear regression analysis determines statistical significance (FDR < 0.001, *R*^*2*^ = 0.0893 and *R*^*2*^ = 0.6118)

Global 5hmC levels are highly variable depending on tissue of origin and developmental stage. The regional distribution of 5hmC is highly tissue-specific, and 5hmC is enriched at regulatory regions of highly transcribed genes in glioblastoma [37–39]. Thus, we explored differentially hydroxymethylated genomic regions (DhMRs) using 16 MB primary tissues and 6 age-matched normal cerebella (Additional file 3: Table S2) by employing a previously established chemical labeling and affinity purification method coupled with high-throughput sequencing [10]. Principal component analysis showed substantial similarity of 5hmC patterns in normal cerebella (*n* = 6) regardless of age (mean age = 11.5 years old, range = 5 to 19 years old), but tumors (*n* = 16, mean age = 9.4 years old, range = 1.5 to 34 years old) showed divergent 5hmC patterns (Additional file 1: Fig. S2). We identified 24,006 DhMRs that show increased hydroxymethylation in MBs (gain-of-5hmC) and 85,738 DhMRs that show decreased hydroxymethylation in MBs (loss-of-5hmC) using DESeq2 [40] (Fig. 2a and Additional file 4: Table S3). Gain-of-5hmC showed significant enrichment at chromosome 2, 15, and 20, whereas loss-of-5hmC was mainly enriched at chromosome 1 and 19 (Additional file 1: Fig. S2). Given the significant association between elevated C to G transversions with asymmetrically hydroxymethylated sites in cancer genomes [41], further investigation is needed to determine the direct correlation between 5hmC alterations and mutagenic events in MBs.

### Gain-of-5hmC is enriched at regulatory regions of genes involved in stem-like properties

Recent studies have revealed significant enrichment of 5hmC at gene bodies of actively transcribed genes in embryonic stem cells (ESCs) and cis-regulatory regions including promoter and enhancer regions [42–45]. Interestingly, gain-of-5hmC was significantly enriched at promoter regions (log2 enrichment = 1.512, *p* < 0.001) and transcription start sites (log2 enrichment = 1.657, *p* < 0.001), whereas loss-of-5hmC was enriched at genic regions including exons (log2 enrichment = 1.146, *p* < 0.001) and introns (log2 enrichment = 0.721, *p* < 0.001), indicating that gain-of-5hmC can play a more active role in gene transcriptional regulation in MBs (Fig. 2b and Additional file 5: Table S4). Notably, 5hmC signals in tumors were highly enriched at previously reported MB enhancer regions [46], while those of normal samples were more evenly dispersed across the genome (Fig. 2c). Moreover, motif analysis with Hypergeometric Optimization of Motif EnRichment (HOMER) [47] identified that gain-of-5hmC was enriched at over 40% of target sequences with *Nanog* and *Scl* (stem cell leukemia) motifs (FDR < 0.05, Fig. 2d and Additional file 6: Table S5). In mouse ESCs, the physical interaction between NANOG and TET1/TET2 proteins yields an increase in 5hmC levels at target regions such as *Esrrb* (estrogen related receptor beta) and *Oct4* (octamer-binding transcription factor 4), elevating the expression of key reprogramming genes [48, 49], suggesting that Nanog can contribute to regional 5hmC formation in MBs. In addition, gain-of-5hmC was highly enriched at other bHLH (basic helix-loop-helix) transcription factors including OLIG2 (oligodendrocyte transcription factor, 20.81%), NEUROG2 (neurogenin 2, 16.17%), and ASCL1 (achaete-scute family BHLH transcription factor 1, 13.97%) (Additional file 6: Table S5), which are involved in neurogenesis and maintenance of stem cell-like properties [50–52]. Gain-of-5hmC was also found in about 15% of binding sites of LIM homeobox genes (LHX1, LHX2, and LHX3), suggesting that gain-of-5hmC has the potential to regulate super-enhancer regions in MBs [46]. Further functional prediction of cis-regulatory regions using Genomic Regions Enrichment of Annotations Tool (GREAT) identified that gain-of-5hmC was enriched at genes involved in cerebellar development, including netrin-mediated signaling, reelin signaling, and genes involved in axon guidance as well as the Notch signaling pathway (Fig. 2e and Additional file 7: Table S6). To investigate whether DhMRs in MBs are indeed involved in controlling stem-like properties, we compared genome-scale patterns of gain-of-5hmC and loss-of-5hmC with normal fetal or adult DhMRs that were published previously [53]. Genomic loci containing 5hmC in fetal samples tended to map to gain-of-5hmC regions although many 5hmC regions associated with MBs still overlapped with those of adult samples (*R*^*2*^ = 0.0893, FDR < 0.001, Fig. 2f). In addition, 5hmC regions from adult substantially mapped to loss-of-5hmC regions (*R*^*2*^ = 0.6118, FDR < 0.001, Fig. 2g). Collectively, these results show that 5hmC patterns in human MBs contain distinct 5hmC features of both fetal and adult cerebellum, suggesting that tumor cells might coopt or retain fetal epigenomic methylation patterns that promote stemness during tumorigenesis.

### The 5hmC signature of MBs in SmoA1 mice recapitulates that of human MB

**Fig. 3:**
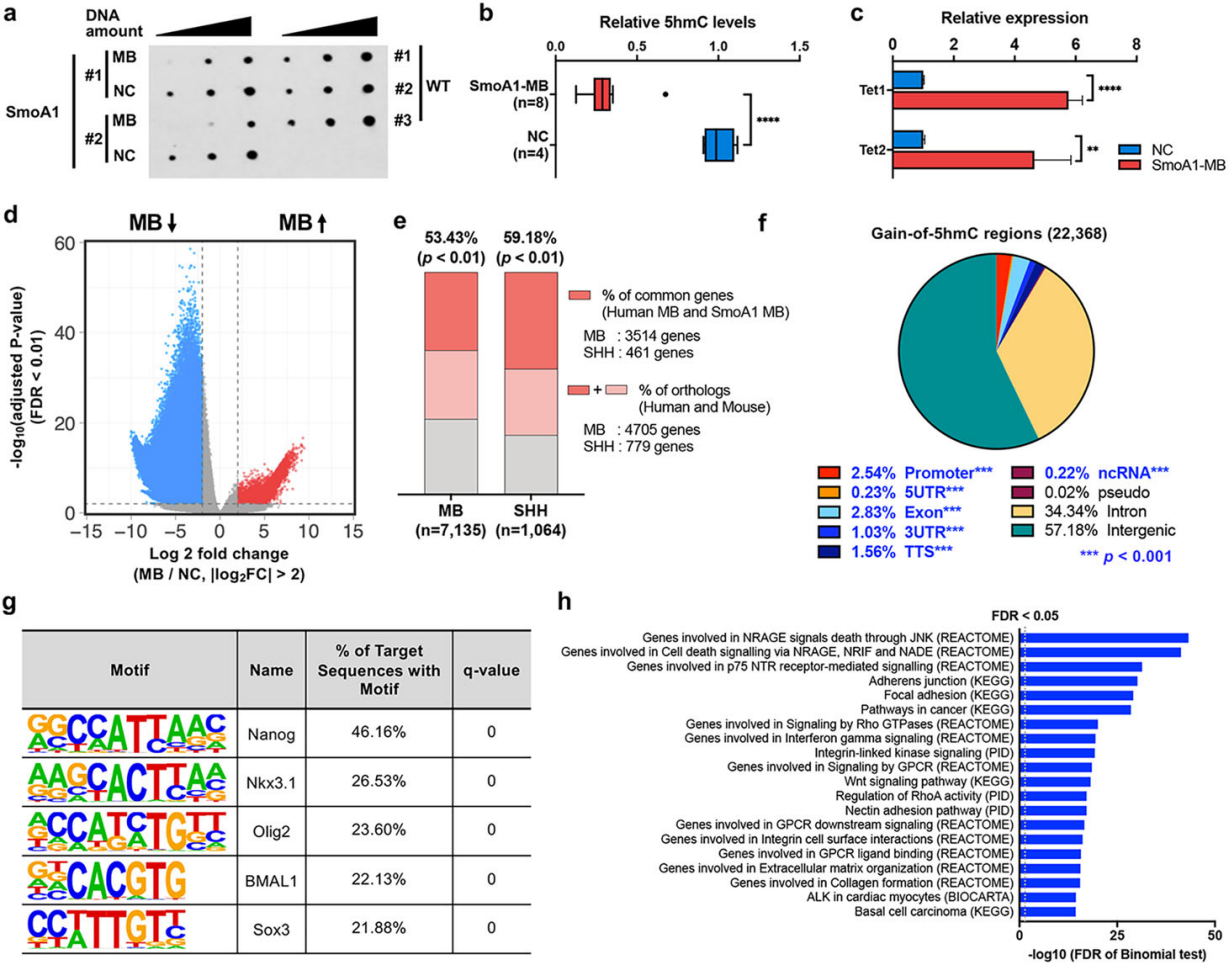
5hmC signature in SmoA1 mouse model recapitulates human MB signature. **a** 5hmC dot blot analysis using SmoA1-MBs (*n* = 4) and surrounding NCs (*n* = 4). **b** Boxplot showing relative 5hmC levels in SmoA1-MBs (*n* = 8) and NCs (*n* = 4) (****p < 0.001). **c** Relative expression level of *Tet1* and *Tet2* in SmoA1-MBs compared to NC. Each region is normalized using *Gapdh* signal (****p < 0.001 and **p < 0.01). **d** Volcano plot showing the hMe-seal data of 4 SmoA1-MB and 5 NC samples. Red dots indicate gain-of-5hmC regions in SmoA1-MB, and blue dots indicate loss-of-5hmC in SmoA1-MB. The same binning and criteria used in hMe-seal data analysis for human MB samples were applied. **e** Bar graphs displaying commonly identified genes in both human MBs (either 16 MBs or 4 SHH-MBs) and SmoA1-MBs (*n* = 4). Gray indicates genes associated with human MBs (*n* = 2430) and SHH-MBs (*n* = 285) only, pink indicates genes which have mouse orthologs (human MBs *n* = 1191, SHH-MBs *n* = 318) but were not identified in mouse MBs, and red indicates genes which are commonly identified in both human and mouse MBs (human MBs *n* = 3514, SHH-MBs *n* = 461). Mouse orthologs from both human MBs and SHH-MBs are significantly identified in SmoA1-MBs and the percentage are indicated above each bar (*p* < 0.01). **f** Pie charts illustrating the annotation summary of 5hmC gain in SmoA1-MB (*n* = 22,368) using HOMER. Annotations with FDR < 0.001 compared to background are highlighted in blue. **g** Sequence logos are shown for the highly enriched sequence motifs in 5hmC acquiring genomic regions in SmoA1-MB. **h** Top 20 pathways identified by Molecular Signatures Database (MSigDB) enrichment analysis with GREAT using 5hmC gain in SmoA1-MB. All statistical tests were performed using the same parameters used in human MB data analysis

To further explore the role of 5hmC and TET in MB progression, we utilized the *SmoA1* mouse model, as 5hmC signatures are not maintained in cell culture mainly due to a drop in TET expression [23, 37]. These mice constitutively express active *Smo*, specifically in granule neuron precursors (GNPs) and are prone to spontaneous MB development in the cerebellum [54, 55]. Consistent with human MB, global 5hmC levels were significantly reduced (Fig. 3a, b and Additional file 1: Fig. S3a) and *Tet1* and *Tet2* gene expression increased (Fig. 3c) in the transgenic mice tumors, compared to adjacent normal cerebellum. Profiling of the genome-wide 5hmC distribution in 4 mouse MBs compared to 5 normal cerebellum samples showed distinct patterns in tumors (Additional file 1: Fig. S3), and we identified 22,368 gain-of-5hmC regions and 317,120 loss-of-5hmC regions (Fig. 3d and Additional file 8: Table S7). Next, we looked for similarities between the two species’ tumors by comparing the conserved regions of gain-of-5hmC. We performed this analysis using either all human MB groups combined (*n* = 16) or focusing only on human MBs classified into the SHH subgroup (*n* = 4) since the *SmoA1* mouse model was developed to produce mice with a high incidence of Hedgehog (Hh) signaling associated MBs, which mimics SHH-MB patients. We identified an additional 1352 gain-of-5hmC regions in the 4 SHH-MBs (Additional file 1: Fig. S3e. We then found conservation rates to the mouse genome using the LiftOver tool (UCSC) with all gain-of-5hmC regions of human MBs and SHH-MBs, which were 99.5% and 94.0%, respectively (Additional file 1: Fig. S3 and Additional file 9: Table S8). In total, 21.9% of the 1257 conserved gain-of-5hmC regions of SHH-MBs were commonly identified in gain-of-5hmC regions of SmoA1-MBs (Fisher’s exact test *p* < 0.01, Additional file 1: Fig. S3e and Additional file 9: Table S8). In addition, a substantial number of the 22,254 conserved gain-of-5hmC regions of all human MB groups were detected in gain-of-5hmC regions of SmoA1-MBs; however, the percentage of overlap between all human MBs and SmoA1-MBs was smaller (5.18%; Fisher’s exact test *p* < 0.01, Additional file 1: Fig. S3f and Additional file 9: Table S8). Given that the context-dependent 5hmC signature may not be well conserved between species, and 5hmC plays a role in gene expression, we also explored how many genes near gain-of-5hmC regions of either human MBs (*n* = 16) or SHH-MBs (*n* = 4) were overlapped with the 9051 genes near gain-of-5hmC regions of SmoA1-MBs (Fig. 3e, Additional file 1: Fig. S3g). Interestingly, 53.43% of mouse orthologs of genes near gain-of-5hmC regions of all human MBs (mouse orthologs = 4705, total = 7135) were commonly identified in genes near gain-of-5hmC regions of SmoA1-MBs, which was comparable with the overlapping percentage (59.18%) of mouse orthologs of genes near gain-of-5hmC regions of SHH-MBs (mouse orthologs = 461, total = 1064) (Fisher’s exact test *p* < 0.01, Fig. 3e, Additional file 1: Fig. S3g and Additional file 10: Table S9). In addition, we performed similar analyses using loss-of-5hmC regions. Interestingly, we also identified substantial overlap of loss-of-5hmC between human MB and mouse in the genomic level (54.20%) and gene level (84.43%) (Additional file 1: Fig. S3h), indicating that both gain-of-5hmC and loss-of-5hmC could be cancer-specific epigenetic marks.

Consistent with human annotation features, gain-of-5hmC regions of SmoA1-MB were enriched at promoter regions (log2 enrichment = 1.228, *p* < 0.001) and transcriptional start sites (log2 enrichment = 1.764, *p* < 0.001) (Fig. 3f and Additional file 11: Table S10), while loss-of-5hmC regions of SmoA1-MB were enriched at intron (log2 enrichment = 0.784, *p* < 0.05) and 3′UTR regions (log2 enrichment = 0.806, *p* < 0.05, Additional file 1: Fig. S3i). Motif analysis and functional prediction of cis-regulatory regions using GREAT analysis revealed high concordance with the 5hmC signature identified in human MBs (Fig. 3g, h, and Additional file 12: Table S11 and Additional file 13: Table S12). Altogether, these data show strong epigenetic similarities between the SmoA1-derived MBs and human MBs, regardless of molecular grouping.

### TET1 is a key enzyme in modulating MB progression

**Fig. 4:**
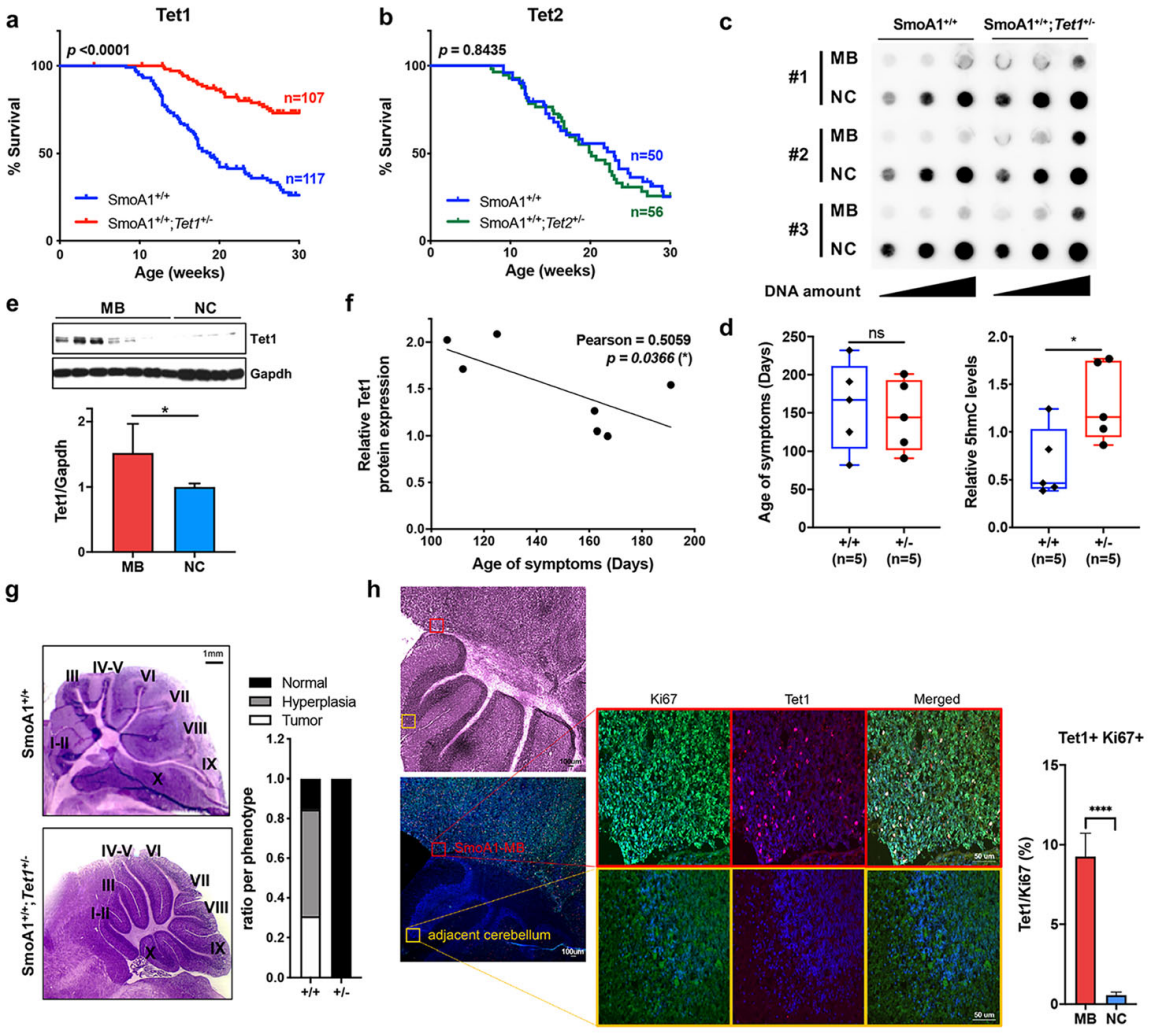
Elevated Tet1 is essential for MB progression. **a**, **b** Kaplan–Meier curves show the significant increase in survival from SmoA1^+/+^ mice crossed with hemizygous deletion of *Tet1* (**a** *p* < 0.0001; log-rank test), but not crossed with hemizygous deletion of *Tet2* (**b** *p* = 0.5830; log-rank test). **c**, **d** 5hmC dot blot analysis shows significant increase in 5hmC levels in tumors from SmoA1^+/+^;*Tet1*^+/−^ mice (*n* = 5; 3 representative samples shown) compared to tumors from SmoA1^+/+^ (*n* = 5; 3 representative samples shown) despite similar ages of tumor-associated symptoms shown in box plot below dot blot (*n* = 5 per group). **e** Tet1 protein expression is significantly higher in SmoA1-MBs (*n* = 7) compared to corresponding NCs (*n* = 4) (*p* < 0.05). **f** Pearson correlation between Tet1 expression and age-of-onset (Pearson *R*^*2*^ = 0.5059, *p = 0.0366). **g** H&E staining (left) and ratio per phenotype (right) of 12-week-old SmoA1^+/+^ mice in the presence of either wild-type or hemizygous deletion of *Tet1* (*p* < 0.0001; Welch’s *t*-test). **h** Fluorescence microscopy of normal cerebellum and MB with Ki67 (red) and Tet1 (green) in 12-week-old SmoA1^+/+^ mice showing Tet1 expression is significantly higher in Ki67-positive cells (blue: DAPI, **** *p* < 0.001)

As *TET1* and *TET2* were consistently overexpressed in both human and murine MBs (Figs. 1e and 3c), we examined whether loss of either may alter the formation and progression of murine MBs. Since mice bearing MBs show distinguishable features including hunched posture and typically die within 2 weeks due to difficulty with eating [54, 55], we investigated onset of medulloblastoma-associated symptoms. Interestingly, crossing *SmoA1*^*+/+*^ mice with *Tet1* knockout mice led to a dramatic delay in age-of-onset of medulloblastoma-associated symptoms in *SmoA1*^+/+^;*Tet1*^+/−^ (median survival 18.86 weeks versus undetermined) and a decrease in incidence of MB (42.7% versus 82.2% at 20 weeks; *p* < 0.0001; log-rank test) (Fig. 4a, Additional file 1: Fig. S4a), while no significant effects were observed upon crossing with *Tet2* knockout mice (*p* = 0.8435; log-rank test, Fig. 4b, Additional file 1: Fig. S4a). The effect was gene dosage-dependent as homozygous loss of *Tet1* further reduced penetrance, particularly on a heterozygous *SmoA1* background (Additional file 1: Fig. S4b). MBs derived from *SmoA1*^+/+^;*Tet1*^+/−^ mice showed significantly higher global 5hmC levels than tumors from *SmoA1*^*+/+*^ compared to mice with similar age of disease-related symptoms (Fig. 4c, d and Additional file 1: Fig. S4c), indicating that TET1 is a major contributor of global 5hmC depletion found in MBs. We then determined the correlation between TET1 expression and age-of-onset. Consistent with mRNA expression levels, TET1 protein levels were significantly elevated in MBs (*p* < 0.05; Fig. 4e) and exhibited a significant inverse correlation with age-of-onset (*R*^*2*^ = 0.5059, *p* = 0.0366; Fig. 4f). Histological examination revealed a high incidence of hyperplasia (< 50% of the cerebellum morphologically abnormal) and invasive tumors (> 50% of the cerebellum morphologically abnormal) in 12-week-old *SmoA1*^+/+^ mice which contained higher Ki67 positive cells (Fig. 4g, Additional file 1: Fig. S4), but we observed normal cerebellar morphology from the majority of *SmoA1*^*+/+*^*;Tet1*^+/−^ mice (Fig. 4g, Additional file 1: Fig. S4d). Immunofluorescence staining data showed TET1 expression is significantly higher in Ki67 positive cells (Fig. 4h). Taken together, these results demonstrate that TET1, not TET2, is a key TET enzyme involved in abnormal proliferation in MB progression.

### Inhibition of TET1 expression in MB cells attenuates tumor growth in vitro

**Fig. 5:**
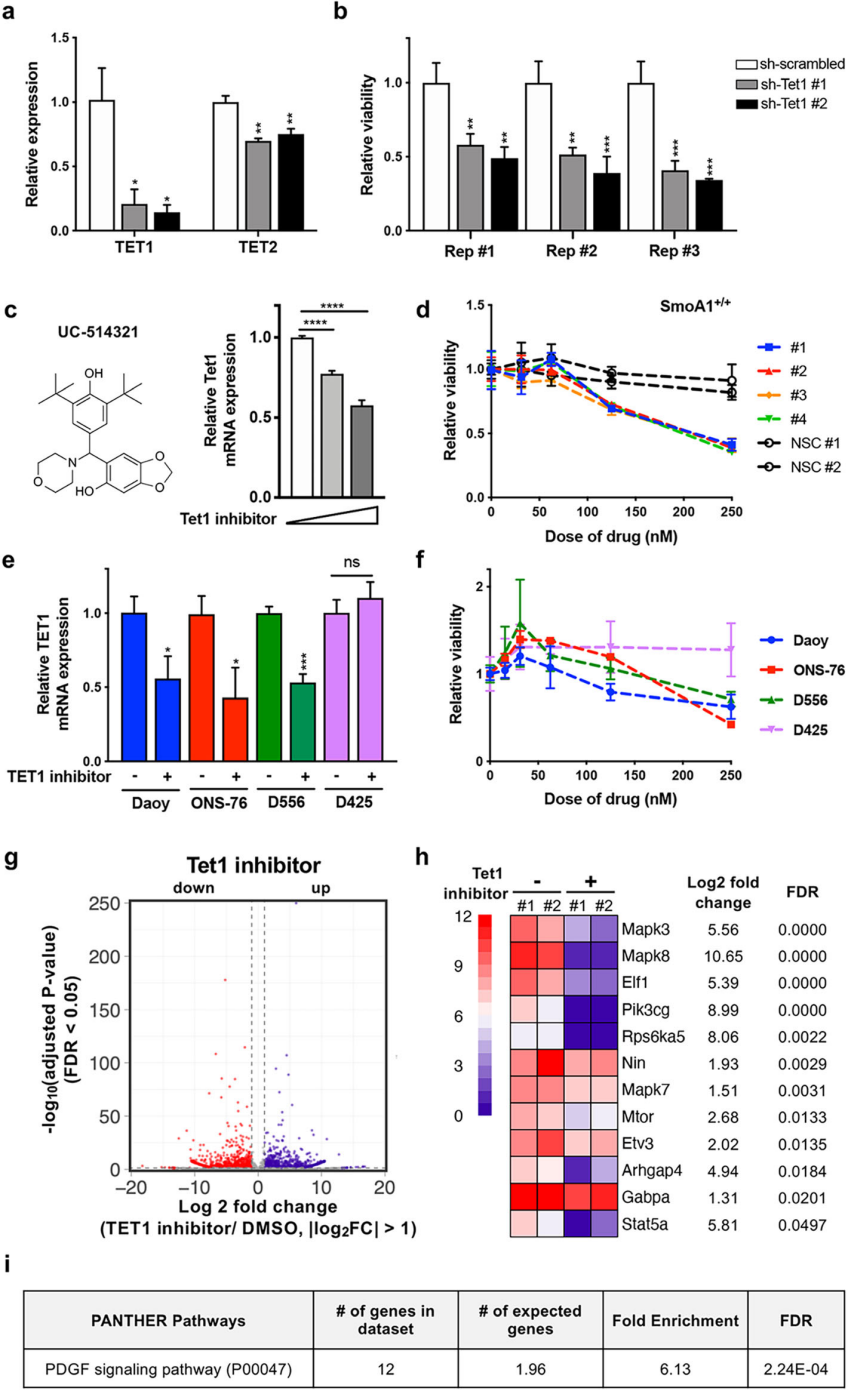
TET1 inhibition confers cytotoxic effect on both SmoA1- and human MBs. **a** mRNA *Tet1* and *Tet2* expression upon shRNA treatment targeting *Tet1* in primary cultures of SmoA1-MBs. Expression was normalized with *Gapdh* expression (* indicates *p* < 0.05 and ** indicates *p* < 0.01). **b** Relative cell viability in 5 days of two different sh-Tet1-treated primary cells compared to sh-scrambled-treated primary cells for three biological replicates (** indicates *p* < 0.01 and *** indicates *p* < 0.001). **c** Left: structure of TET1 inhibitor UC-514321. Right: dose-dependent expression of *Tet1* 2 days after chemical treatment (0 nM, 100 nM, and 200 nM, respectively, *p* < 0.0001 for both). **d** Relative cell viability depending on dose of drug (nM) in SmoA1^+/+^. NSC: Neuronal stem cell. **e** mRNA *Tet1* expression upon 200 nM of UC-514321 treatment in MB cell lines, including Daoy (*p* < 0.05), ONS-76 (*p* < 0.05), D556 (*p* < 0.001), and D425 (no significance). **f** Relative cell viability depending on dose of drug (nM) in MB cell lines. **g** Volcano plot showing RNA-seq data of 2 DMSO-treated and drug-treated primary cultures of SmoA1-MBs. Purple dots indicate upregulated transcripts, and red dots indicate downregulated transcripts 2 days after TET1 inhibitor treatment. Transcripts with absolute fold change > 2 and adjusted *p* value < 0.05 are considered statistically significant. **h** Genes significantly downregulated after TET1 inhibitor treatment (absolute fold change > 2 and FDR < 0.05). **i** PANTHER pathway analysis using downregulated genes after TET1 inhibitor treatment

To determine whether abrogation of *Tet1* expression attenuates tumor cell growth, we used small hairpin RNA (shRNA) targeting of *Tet1* in primary cell cultures of SmoA1-MBs. The knockdown was specific to *Tet1* and resulted in a 50% decrease in cell viability (Fig. 5a, b), while the overexpression of TET1 rescued the knockdown phenotype (Additional file 1: Fig. S5a, b). To further investigate whether pharmacological inhibition of TET1 could also have the same impact on MB cells, we used UC-514321, a small molecule that suppresses the expression of *Tet1* by inhibiting binding of STAT (signal transducer and activator of transcription) transcription factors (STAT3 and STAT5) at *Tet1* promoter regions [56]. SmoA1-derived MBs showed higher expression of STAT3 as well as higher levels of phosphorylated STAT3 while STAT5 level was slightly increased (Additional file 1: Fig. S5c, d, e), implying that STAT transcription factors may be a major contributor to *Tet1* upregulation in MBs. Consistent with shRNA-mediated *Tet1* inhibition, we observed a dose-dependent cytotoxic effect of the inhibitor in primary cell cultures of SmoA1-MBs, but not in normal neuronal stem cells (NSCs) (Fig. 5c, d and Additional file 1: Fig. S5f), indicating that this inhibitor can selectively induce cell death of tumors. The pharmacological benefit of UC-514321 observed in SmoA1-MBs was also reproduced in human MB cell lines (Fig. 5e, f and Additional file 1: Fig. S5f). Notably, only TET1-expressing lines (Daoy, ONS-76, and D556) were responsive to the inhibitor; no effect was seen in the non-TET1-expressing line (D425) (Fig. 5e, f and Additional file 1: Fig. S5g). To further investigate which genes and signaling pathways were significantly altered upon inhibitor treatment, we performed RNA-seq analysis (Fig. 5g). Intriguingly, mitogen-activated protein kinases (*Mapk3*, *Mapk8*, and *Mapk7*) and phosphoinositide 3-kinases (*Pik3cg)* were significantly downregulated with the TET1 inhibitor treatment (Fig. 5h and Additional file 14: Table S13), and PANTHER pathway analysis identified significant enrichment of PDGF signaling pathway (Fig. 5i). Both shRNA treatment and TET1 inhibitor data indicate that targeting *Tet1* has the potential to suppress MB growth.

### TET1 contributes to Pdgfr upregulation through interacting with Nanog

**Fig. 6:**
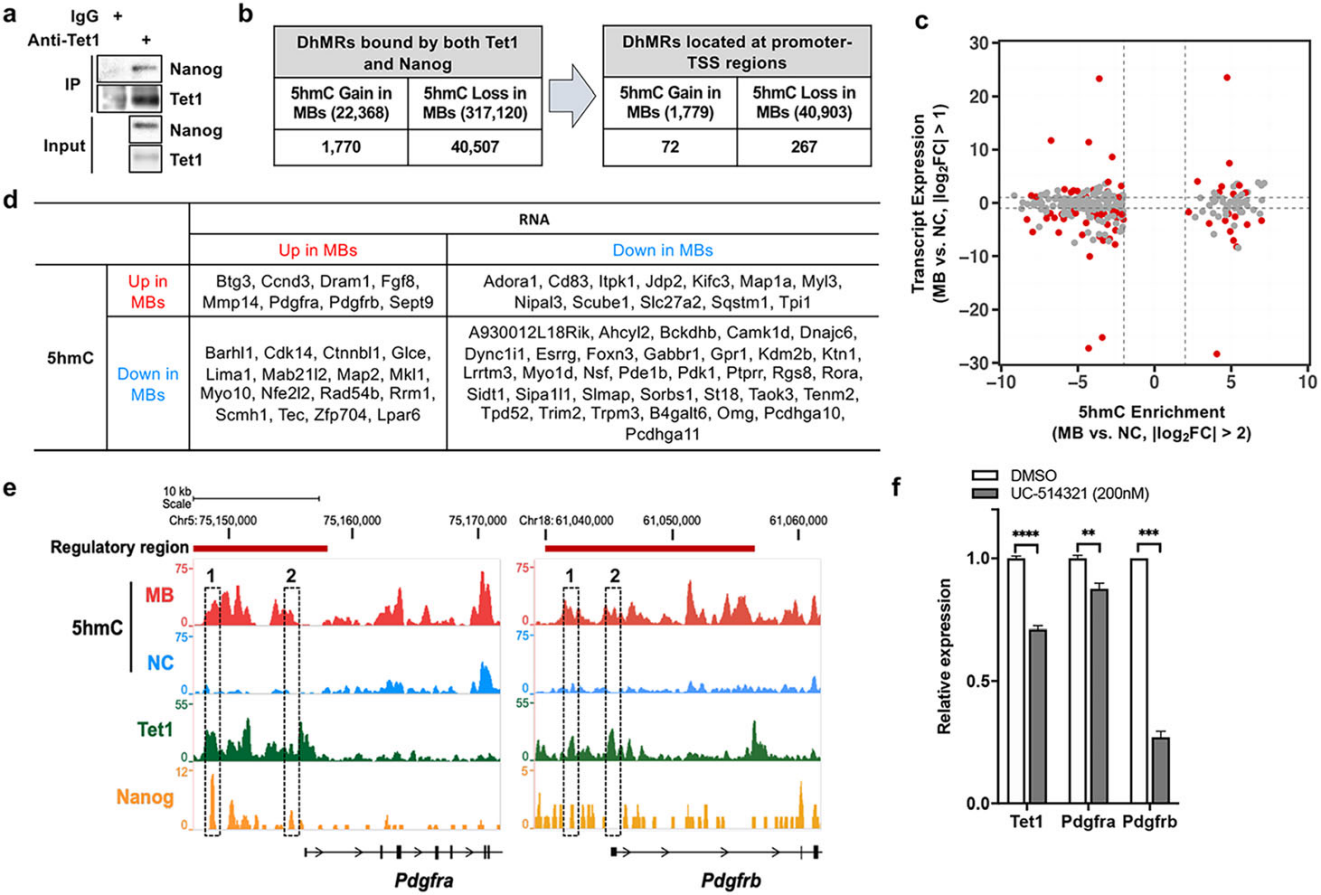
Pdgfrs are potential downstream targets of Tet1. **a** Co-immunoprecipitation of Nanog with Tet1 protein in SmoA1-MB. **b** Stepwise identification of DhMRs bound by Tet1 and Nanog as well as located at promoter-TSS regions. **c** Scatterplot illustrating the expression of transcripts with either gain-of-5hmC or loss-of-5hmC at their promoter-TSS regions. Red dots indicate transcripts significantly either upregulated or downregulated in SmoA1-MB. **d** Summary table of genes with differential 5hmC levels at their promoter-TSS regions and gene expression. **e** Peaks of Tet1 and Nanog from ChIP-Seq studies [57–59] and 5hmC peaks from MBs and NCs in the *Pdgfra* and *Pdgfrb* loci. The loci highlighted by dotted rectangles were further validated by TET1 ChIP-qPCR. **f** Both *Pdgfra* and *Pdgfrb* expressions are downregulated upon TET1 inhibitor treatment (**p < 0.01, ***p < 0.001, and ***p < 0.0001)

Substantial enrichment of gain-of-5hmC at NANOG binding sites was identified in both human and murine MBs (48.84% and 46.16%, respectively), and NANOG is a known binding partner of TET1 in the mouse ESCs [48, 49]. We also detected an interaction between TET1 and NANOG in mouse SmoA1-MBs (Fig. 6a). Therefore, we performed a meta-analysis of previously identified genomic regions bound by both NANOG and TET1 [48] and our hMe-seal sequencing and RNA-sequencing data. We first identified the DhMRs which are located at binding regions of both TET1 and NANOG (Fig. 6b), and selected the regions located at promoter-TSS regions (Fig. 6b and Additional file 15: Table S14). In total, 71 gain-of-5hmC regions (corresponding to 70 unique genes) and 266 loss-of-5hmC regions (corresponding to 227 unique genes) were identified in MB DhMRs (Additional file 15: Table S14). We then examined gene expression changes in 297 identified genes and identified eight genes (*Btg3*, *Ccnd3*, *Dram1*, *Fgf8*, *Mmp14*, *Pdgfra*, *Pdgfrb*, and *Sept9*) which had higher 5hmC signals at their promoter regions and showed higher expression in SmoA1-MBs (Fig. 6c, d). Among these eight genes, genomic alterations in *Ccnd3*, *Pdgfra*, *Pdgfrb*, and *Sept9* are known for their implication in tumorigenesis in multiple cancer types [60]. PDGFR subunits -a and -b are well-known cell surface tyrosine kinase receptors implicated in cell proliferation, growth, and development. In metastatic MBs, both PDGFRs are highly overexpressed compared to non-metastatic tumors and are involved in the regulation of genes in the RAS/MAPK signal transduction pathway [61, 62]. We performed a chromatin immunoprecipitation (ChIP) assay using SmoA1-MB and found that TET1 could bind to the regulatory region of either *Pdgfra* or *Pdgfrb* where 5hmC levels are significantly elevated (Fig. 6e and Additional file 1: Fig. S5h). Interestingly, some of TET1 binding overlapped with NANOG binding, suggesting that NANOG, which interacts with TET1 in SmoA1-MB, can bind to the same regions along with TET1. To further determine whether the inhibition of *Tet1* expression leads to a decrease in *Pdgfra* and *Pdgfrb* expression, we treated primary cell cultures of SmoA1-MBs with the TET1 inhibitor and identified that both *Pdgfra* and *Pdgfrb* were downregulated (Fig. 6f). In summary, these data indicate that PDGFRs are downstream targets of TET1 and NANOG, their expression can be regulated by a TET1 inhibitor, and thus, TET1 is a potential therapeutic target to treat TET1-overexpressing MBs.

## Discussion

MBs, the most aggressive pediatric brain tumors, can be subdivided into four molecular groups based on transcriptome and methylation profiling: WNT, SHH, Group 3, and Group 4 [63]. Recent studies, however, have demonstrated that the current molecular grouping is insufficient to predict patients’ prognosis. For instance, tumor protein P53 (*TP53)* mutation is a critical risk factor in both WNT and SHH-MB subgroups [64]. In addition, aberrant DNA methylation at the genes involved in embryonic morphogenesis is also considered as a potential driver of the oncogenic process in the SHH subgroup diagnosed after 5 years of age (MB_SHH-Children_) [65]. Since loss of 5hmC, the first oxidative derivative of 5mC, has served as an unfavorable indicator for several malignant tumors (e.g., high-grade glioma (GBM) and melanoma [13, 66]), we investigated alteration of 5hmC in MB. Consistent with prior findings in other tumor types, we have identified a significant reduction in 5hmC across MB genomes and a strong inverse correlation between 5hmC levels and prognosis. Importantly, we identified the enrichment of an MB-specific 5hmC signature at NANOG, a key transcription factor involved in self-renewal and pluripotency of ESCs. NANOG is overexpressed in MBs, which prevents differentiation and maintains stemness of tumors [67]. Moreover, compared to normal tissues, 5hmC signals in MBs were higher at the MB active enhancers of cis-acting transcription activating elements, suggesting that 5hmC plays a critical role in regulating MB-associated gene expression. We also identified that gain-of-5hmC regions were involved in the activation of embryonic development signaling pathways such as the Notch signaling pathway. The activation of Notch signaling not only induces expression of stem-like markers and cell growth in tumors but also confers drug resistance through multidrug resistance ABC (ATP-binding cassette) transporters [68, 69]. Indeed, the resemblance of gain-of-5hmC regions to fetal 5hmC patterns indicates that gain-of-5hmC plays a critical role in maintaining stem cell-like properties in MBs.

Ten-eleven translocation (TET) enzymes (TET1, TET2, and TET3) are α-ketoglutarate-dependent dioxygenases that convert 5mC to 5hmC [70, 71], and therefore, their expression tends to be strongly correlated with 5hmC levels. Indeed, the overexpression of TET2 in melanoma cells suppresses tumor initiation and progression by increasing 5hmC levels [72], and elevated 5hmC by upregulated TET1 recruits the CHOIP-methylosome complex near the genes involved in glioblastomagenesis [73]. Surprisingly, we observed an overall loss of 5hmC and elevated TET1 and TET2 in clinical MB samples. Given the frequent identification of TET loss of function mutations along with global hypomethylation, regional hypermethylation in cancer, and the redundant actions of TET proteins in terms of 5hmC formation [74, 75], the abnormal expression of TET proteins may not always show correlation with global 5hmC levels. In addition, when crossing *Tet1*^+/−^ mice with the *SmoA1* mouse model, which has a high incidence of spontaneous MB development, we found a dramatic decrease in tumor incidence and tumor onset while the abolishment of *Tet2* did not change tumor incidence and age-of-onset. In addition, shRNA- and chemically mediated downregulation of *Tet1* promoted cell death in both murine and human tumors. Further investigation identified *Pdgfra* and *Pdgfrb* as potential downstream targets regulated by TET1 and NANOG. The regulatory regions of *Pdgfra* and *Pdgfrb* are not only binding sites for TET1 and NANOG, but also show high levels of 5hmC in MBs compared to NCs. In addition, a TET1 inhibitor significantly decreased *Pdgfr*s’ expression. Although additional investigation is needed to determine whether TET1 is a bona fide oncoprotein in MB tumorigenesis and whether overexpressed TET2 is cooperative with TET1 to establish global 5hmC level during MB tumorigenesis, our genetic studies demonstrate that TET1 modulates MB tumorigenesis by tumor-specific 5hmC formation along with NANOG. In addition, further investigation to demonstrate the relationship of overexpressed TET1 and tumor-specific 5hmC signature is needed.

## Conclusions

We present the first comprehensive genome-wide profiling of 5hmC and its role in maintaining stemness in MB tumorigenesis. We also identify *TET1* as a heretofore unknown MB oncogene and its role in tumor formation. Our data further show that small molecule-mediated suppression of *TET1* can be a therapeutic option for MB subgroups having highly expressed *TET1*. These findings provide an insight into a new epigenetic driver, “epi-driver,” in pediatric brain tumor and the biological importance of the driver in tumor-associated signaling pathway.

## Methods

All experimental methods comply with the Helsinki declaration.

### Human tissues

Medulloblastoma (MB) samples were collected from three different sources: Aflac cancer center (*n* = 5), the Xiangya Hospital Department of Neurosurgery (*n* = 24), and Dr. Erwin G. Van Meir (*n* = 8). UHPLC-MS/MS assay used in Fig. 1a and Additional file 1 included 24 MB samples from the Xiangya Hospital and 5 MB samples from Aflac cancer center. For 5hmC profiling in Figs. 2 and 3, MB samples from Xiangya, 5 MB samples from Aflac cancer center, and 8 MB samples from Dr. Erwin G. Van Meir were used. The patient data was anonymized prior to use in this study. For MB tissue samples from Aflac cancer center, molecular subgroup affiliation was determined by NanoString nCounter system using 22 MB subgroup-specific gene expression profiles. The protocols were approved by the Institutional Review Board at Emory University. Twenty-four MB tissue samples range from 3 to 18 years old with average-risk (children older than 3 years of age with no evidence of metastatic disease and less than 1.5 cm^3^ of residual disease) were collected at the Department of Neurosurgery of Xiangya Hospital. All participants or their legal guardians enrolled in this study provided informed consent. Five normal cerebellum samples used in Fig. 1a as normal control for dot blot assay were collected from patients from 3 to 18 years old with cerebral injury who underwent internal decompression. Six normal cerebellum samples from 5 to 19 years old with no neurological disorders were collected from the NIH NeuroBioBank tissue repositories, which were used as normal control for the dot blot assay (Additional file 1: Fig. S1) and genome-wide 5hmC profiling (Fig. 2).

### Mice

All protocols for mouse experiments were approved by the Institutional Animal Care and Use Committee (IACUC) at Emory University. *Tet1*^+/−^ and *Tet2*^+/−^ mice [76, 77] were initially on a mixed C57BL/6x129S4/Sv background and were backcrossed with WT C57BL/6 mice for more than 10 generations before any of the experiments in this paper. SmoA1 homozygous mice [55] were crossed with either *Tet1*^+/−^ mice or *Tet2*^+/−^ to generate cohorts in this study. Mice were humanely euthanized using isoflurane inhalation upon the signs of disease-related symptoms.

### Primary MB culture

Mice were euthanized by isoflurane inhalation when they showed disease-associated symptoms including hunched posture, tilted head, and lethargy. Isolated tumor tissues were minced in sterile HBSS to obtain a single-cell suspension. To remove cell aggregates and extraneous tissue, the suspension was passed through two different size cell strainers (100 μm and 40 μm) and spun down to collect a cell pellet. The cell pellet was resuspended in Neurobasal medium supplemented with B-27 supplements, l-glutamine, sodium pyruvate, and Pen/Strep and plated at 1.5 × 10^6^ cells per well in a 24-well plate on Matrigel-coated wells. For shRNA-Tet1 treatment, wells were infected with lentivirus at a multiplicity of infection (MOI) of 10 and incubated for 5 days. For TET1 inhibitor (UC-51432) treatment [56], wells were incubated with appropriate concentration of the chemical for 48 h.

### Human MB cell line culture

Human MB cell lines (ONS-76, Daoy, D556, and D425) were cultured with DMEM with 10% fetal bovine serum, 100 U/ml penicillin, and 100 mg/ml streptomycin at 37 °C in an atmosphere of 5% CO_2_.

### Analysis of gene expression Array datasets

Gene expression data of 273 human MB samples and 31 human cerebellar tissue samples were obtained from GEO Series accession numbers GSE49243, GSE12992, GSE10327, GSE37418, GSE50161, GSE44971, GSE7307, and GSE3526. Data analyses were performed using the Bioconductor package “simpleaffy” [78]. Briefly, data were normalized using the gcrma algorithm, and then, molecular subgroups of tumor samples unclassified in the previous studies were determined by unsupervised hierarchical clustering based on 1-Pearson correlation. Differential gene expression analysis was performed using the Bioconductor package “limma” [79], and then, volcano plots and boxplots were generated using the Bioconductor package “ggplot2” [80].

### Genomic DNA preparation and dot blot assay

Genomic DNA preparation and the dot blot assay of 5hmC were performed as described previously [53]. DNA purification was performed by phenol-chloroform precipitation and reconstituted in DNase-free water.

### UHPLC-MRM-MS/MS analysis

Genomic DNA was enzymatically digested into single nucleosides by a mixture of DNase I, calf intestinal phosphatase, and snake venom phosphodiesterase I at 37 °C for 12 h. After the enzymes were removed by ultrafiltration, the digested DNA was subjected to UHPLC-MS/MS analysis. The UHPLC-MS/MS analysis was performed on an ultra-high-performance LC system coupled with a QQQ6490 mass spectrometer equipped with a jetstream electrospray ionization source (Agilent, Santa Clara, CA). A reverse-phase Zorbax SB-C18 column (2.1 × 50 mm, 1.8-μm particles) was used for UHPLC separation. The mass spectrometer was operated under positive ionization using multiple reactions monitoring (MRM) mode. All the quantification data are included in Table S15.

### Survival analysis using human samples and mouse model

Survival was measured from the time of initial diagnosis until the date of death due to progression of disease. The survival distribution was estimated using Kaplan–Meier curves. Survival curves were compared by means of the log-rank test. Results were considered statistically significant when the *p* value of the log-rank (Mantel-Cox) test was below 0.01.

### Genome-wide 5hmC profiling (hMe-seal sequencing)

Tumor tissues and matched normal cerebellar tissues were used for hMe-Seal sequencing [10, 81] to identify differential hydroxymethylation regions (DhMRs). For labeling of 5hmC-containing genomic regions, 1 μg of sonicated genomic DNA (100–300 bp) was incubated for 2 h at 37 °C in a 30 μl solution containing 100 μM UDP-6-N_3_-Glu, β-glucosyltransferase (β-GT) and NEB buffer 4. After purification using AMPure XP beads, N_3_-glucose-labeled DNA was incubated for 2 h at 37 °C with the addition of 150 μM dibenzocyclooctyne-modified biotin (click chemistry), and then purified using AMPure XP beads. DNA libraries were generated using NEBNext® Ultra™ II DNA Library Prep Kit, which were then ready for sequencing.

### Analysis of hMe-seal sequencing data to identify DhMRs

Sequencing data were mapped to either human genome, hg19, for human medulloblastoma and age-matched normal cerebella or mouse genome, mm10, for SmoA1 medulloblastoma using bowtie2 [82]. Mapped reads were filtered and sorted using SAMtools [83] and then PCR duplicates were removed using Picard [84]. Binned matrices (bin size 2 kb) were generated using final bam files, and bins of MB samples were adjusted with average of relative 5hmC levels (0.4367 for human MBs and 0.3148 for SmoA1-MBs). Differential hydroxymethylation regions (DhMRs) were identified using DESeq2 with default parameters [40]. FDR < 0.01 and log2 fold change > 2 were considered as a statistical significance. Identified DhMRs were annotated using Homer [47] and CEAS [85]. To understand the biological meaning of DhMRs, we used GREAT with default “Basal plus extension” settings [86]. Enrichment terms with FDR < 0.05 (both region-based binomial and hypergeometric tests) were regarded as statistically significant. For motif scanning on DhMRs and TET1 binding sites, we used Homer software. To understand the similarity between human MBs and SmoA1-MBs, we compared gain-of-5hmC regions as well as genes near identified gain-of-5hmC regions. For common region identification, human gain-of-5hmC regions (hg19) were converted to mouse gain-of-5hmC regions (mm10) using batch coordinate conversion (liftOver), and then common gain-of-5hmC regions were identified using intersectBed (bedtools). For common gene identification, mouse orthologs corresponding to human genes near human gain-of-5hmC regions were identified using BioMart and then compared with genes near mouse gain-of-5hmC regions. Fisher’s exact was used to test whether observed number of peaks or genes is greater than expected for statistical significance.

### Immunoblotting

Tumor and matched normal tissues were collected from euthanized SmoA1 mice with MB-associated symptoms. Tissues were rinsed with ice-cold PBS, homogenized after the addition of radioimmuno-precipitation assay (RIPA) buffer supplemented with protease inhibitor cocktail. After incubation on ice for 20 min, lysates were then centrifuged in a microfuge at 13,000 rpm for 15 min, supernatants were quantified using bicinchoninic acid (BCA) assay, and 50 μg of each sample was loaded in acrylamide gels for TET1, p-STAT3, STAT3, STAT5a, and GAPDH detection. All immunoblotting was repeated at least three times. For quantitative analysis, autoradiographic films were scanned with an Epson 1680 scanner, and the captured image was analyzed with NIH ImageJ software.

### Brain transcardiac perfusion and histology

For histological analysis, 12-week-old mice from different genotype backgrounds were trans-cardially perfused with 4% paraformaldehyde in PBS. Brains collected were post-fixed with 2% paraformaldehyde in PBS overnight, cryoprotected in 20%, then 30% (w/v) sucrose in PBS at 4 °C, and rapidly frozen. Cryostat sections (10 μm) were stained with hematoxylin and eosin according to the previous Cold Spring Harbor protocol [87]. For immunofluorescent staining, Ki67 (Thermo; 14–5698-82) and TET1 (Genetex; GT1462) antibodies were incubated shaking gently in staining solution (0.5% Triton X-100, 5% normal goat serum, phosphate-buffered saline) at 4 °C and Alexa fluor 488 and 568 were used as secondary antibodies, respectively.

### RNA extraction and RT-PCR

RNA was extracted from pellets using Trizol reagent (Thermo Fisher Scientific) according to the manufacturer’s procedure. After Nanodrop quantification of RNA, 1 μg RNA was used to generate cDNA with SuperScript III First-Strand Synthesis System for RT-PCR. Quantitative PCR for mRNA of *Tet1* and *Pdgfra*, and proper internal control (GAPDH for Mouse and Actin for Human) detection was carried out using SYBR green (Thermo Fisher Scientific) and a 7500 Fast Real-Time PCR machine (Applied Biosystems) with an initial denaturing step at 95 °C for 10 min, then 40 cycles of PCR (95 °C for 15 s, 60 °C for 1 min) and a further extension at 60 °C for 10 min. Specific primer sequences are listed in key resources table.

### Proliferation assay

To assess cell viability after treatment of shRNA and TET1 inhibitor, the CellTiter-Blue Cell Viability Assay (Promega) was used. Briefly, 20 μl of solution was added directly to each well 1 h before measurement. The fluorescence was measured using FLUOstar Omega (BMG Labtech) microplate reader. All measurements were taken in triplicate and each experiment was replicated at least three times.

### Analysis of RNA-sequencing data

RNA for sequencing was prepared using Trizol Reagent from primary MB cells treated by either DMSO (control) or TET1 inhibitor (Drug) for 48 h. Raw sequencing data were mapped to mm10 using HiSAT2 and annotated using StringTie [88]. After the identification of gene-level differentially expressed genes using DESeq2 [40], the TPM (transcripts per kilobase million) value of each sample was plotted to make a chart with software GraphPad Prism 8.0 (GraphPad, Inc.). To understand pathway enrichment of differentially expressed genes, PANTHER pathway analysis [89, 90] was performed and *p* < 0.05 was considered as a statistical significance.

### Co-immunoprecipitation

For co-immunoprecipitation assays, tumor tissues were lysed through brief sonication on ice in lysis buffer (20 mM Tris, pH 7.4, 150 mM NaCl, 0.5% Triton X-100, 1 mM EDTA, 10% glycerol, 1 mM phenylmethylsulfonyl fluoride, 10 μg/ml aprotinin, 10 μg/ml leupeptin, 1 mM sodium fluoride, 1 mM sodium orthovanadate, and 25 mM β-glycerophosphate), and then centrifuged at 4 °C at maximum speed for 10 min. One milligram of the supernatant was then incubated with anti-Tet1 (GeneTex) or control IgG overnight at 4 °C with rotation, followed by further incubation with Protein-G beads (Pierce) for 4 h at 4 °C. The following immunoblotting assays were carried out to detect the target proteins as indicated.

### Chromatin Immunoprecipitation (ChIP)

SmoA1-MBs were treated with 1% formaldehyde for 10 min at room temperature with gentle shaking. Fixation was terminated by adding 2 M fresh glycine to reach a 0.125 M final concentration, and then shaking continued for an additional 5 min. The cell pellet was collected by spinning down at 3750 rpm for 10 min at 4 °C. The cell pellet was then resuspended in 1 mL Nuclei Swelling Buffer (10 mM HEPES/pH 7.9, 0.5% NP-40, 1.5 mM MgCl2, 10 mM KCl, 0.5 mM DTT, and protease inhibitor cocktail), incubated on ice for 10 min, and centrifuged at 5000 rpm for 5 min. Nuclear pellets were further lysed in 200 ml SDS lysis buffer (20 mM HEPES/pH 7.9, 25% glycerol, 0.5% NP-40, 0.5% Triton X-100, 0.42 M NaCl, 1.5 mM MgCl_2_, 0.2 mM EDTA), and protease inhibitor cocktail. Cell lysate was sonicated 8 times with 0–3 Power Output for 30 s each (at least 30 s cooling on ice between each 30 s sonication) to obtain DNA fragments between 200 and 500 bp. After sonication, nuclear lysate was cleared by centrifugation at 13,000 rpm for 10 min at 4 °C to collect the supernatant. The nuclear lysate was diluted with 4 volumes of dilution buffer (0.01% SDS, 1% Triton X-100, 1.2 mM EDTA, 167 mM NaCl, 16.7 mM Tris-HCl/pH 8.0, and protease inhibitor cocktail). Diluted nuclear lysate was pre-cleared by 30 ml pre-washed Protein-G Dynabeads (Thermo Fisher, cat# 10009D) for 2 h at 4 °C. After pre-clear, immunoprecipitation was performed with TET1 antibody (GeneTex, GTX627420) with overnight rotation at 4 °C. Thirty milliliters pre-washed Protein-G Dynabeads (Thermo Fisher, cat# 10009D) were added for an additional 2 h. The beads were then washed and eluted. The ChIPed DNA fragments were purified using QIAquick PCR Purification Kit (QIAGEN, cat#28106). ChIPed DNA and input DNA was used for qPCR.

### Meta-analysis to identify genes potentially regulated by TET1

DhMRs bound by TET1 and NANOG as well as located at promoter-TSS regions were identified by two steps: first, TET1- and NANOG-bound DhMRs were detected via intersectBed [91], and then, DhMRs were annotated using HOMER. Total RNA for sequencing was prepared using Trizol Reagent from primary SmoA1-MB tissues and adjacent normal cerebella (NC) followed by data analysis pipeline used for RNA-seq data analysis with drug-treated MB primary cells.

## Supplementary Information

**Additional file 1:** Fig. S1. Loss of 5-hydroxymethylation is a hallmark of MBs. Fig. S2. MB-associated DhMRs are implicated in stem-like properties. Fig. S3. 5hmC signature in the SmoA1 mouse model recapitulates the human MB signature. Fig. S4. Elevated Tet1 is essential for MB progression. Fig. S5. TET1 inhibition confers cytotoxic effect on both SmoA1- and human MBs. Fig. S6. All full western blots in this study.

**Additional file 2:** Table S1. Summary of GEO datasets.

**Additional file 3:** Table S2. MB sample summary.

**Additional file 4:** Table S3. Summary of 5hmC gain and loss regions in human MBs.

**Additional file 5:** Table S4. Summary of annotation using 5hmC gain and 5hmC loss regions.

**Additional file 6:** Table S5. Motif analysis results of human 5hmC gain.

**Additional file 7:** Table S6. Summary of MSigPathway results (GREAT analysis).

**Additional file 8:** Table S7. Summary of 5hmC gain and loss regions in SmoA1-MBs.

**Additional file 9:** Table S8. Summary of similarity between human MBs and SmoA1-MBs (Peaks).

**Additional file 10:** Table S9. Summary of similarity between human MBs and SmoA1 MBs (Genes).

**Additional file 11:** Table S10. Summary of annotation using 5hmC gain and 5hmC loss regions.

**Additional file 12:** Table S11. Motif analysis results of mouse 5hmC gain.

**Additional file 13:** Table S12. Summary of MSigPathway results (GREAT analysis).

**Additional file 14:** Table S13. Summary of transcript ID and gene names identified by RNA-seq.

**Additional file 15:** Table S14. DhMRs bound by both Tet1 and Nanog.

**Additional file 16: **Table S15. Summary of 5hmdC and dC in human normal cerebella (*n* = 5) and human MBs (*n* = 24).

**Additional file 17:** Table S16. Summary of all the key reagents.

**Additional file 18.** Review history.

## Data Availability

Sequencing data have been deposited to GEO with accession number GSE74336 [92] including hMe-seal results for 3 human MB samples and 1 normal sample and the NCBI Sequence Read Archive (SRA) under accession number PRJNA554937 [93] including hMe-seal results for 26 human MBs, 12 NCs, 8 SmoA1-MBs, and 6 NCs, and RNA-seq result for 8 SmoA1-MBs. Human gene expression data were obtained from GEO Series accession numbers GSE49243 [94], GSE12992 [95], GSE10327 [96], GSE37418 [97], GSE50161 [98], GSE44971 [99], GSE7307 [100], and GSE3526 [101]. UHPLC-MS/MS data is available at Figshare with DOI: 10.6084/m9.figshare.14397986.v1 [102]. All the key reagents are listed in Table S16. All the key reagents are listed in Table S16.
