# Supplementary material for: Ten-eleven translocation protein 1 modulates medulloblastoma progression
Source: Genome Biol. 2021 Apr 29;22:125. doi: 10.1186/s13059-021-02352-9 (PMC8082834; doi:10.1186/s13059-021-02352-9)
Supplement: Supplementary file 1 — Additional file 1: Fig. S1. Loss of 5-hydroxymethylation is a hallmark of MBs. Fig. S2. MB-associated DhMRs are implicated in stem-like properties. Fig. S3. 5hmC signature in the SmoA1 mouse model recapitulates the human MB signature. Fig. S4. Elevated Tet1 is essential for MB progression. Fig. S5. TET1 inhibition confers cytotoxic effect on both SmoA1- and human MBs. Fig. S6. All full western blots in this study. [file 13059_2021_2352_MOESM1_ESM.pdf]

ADDITIONAL FILE 1

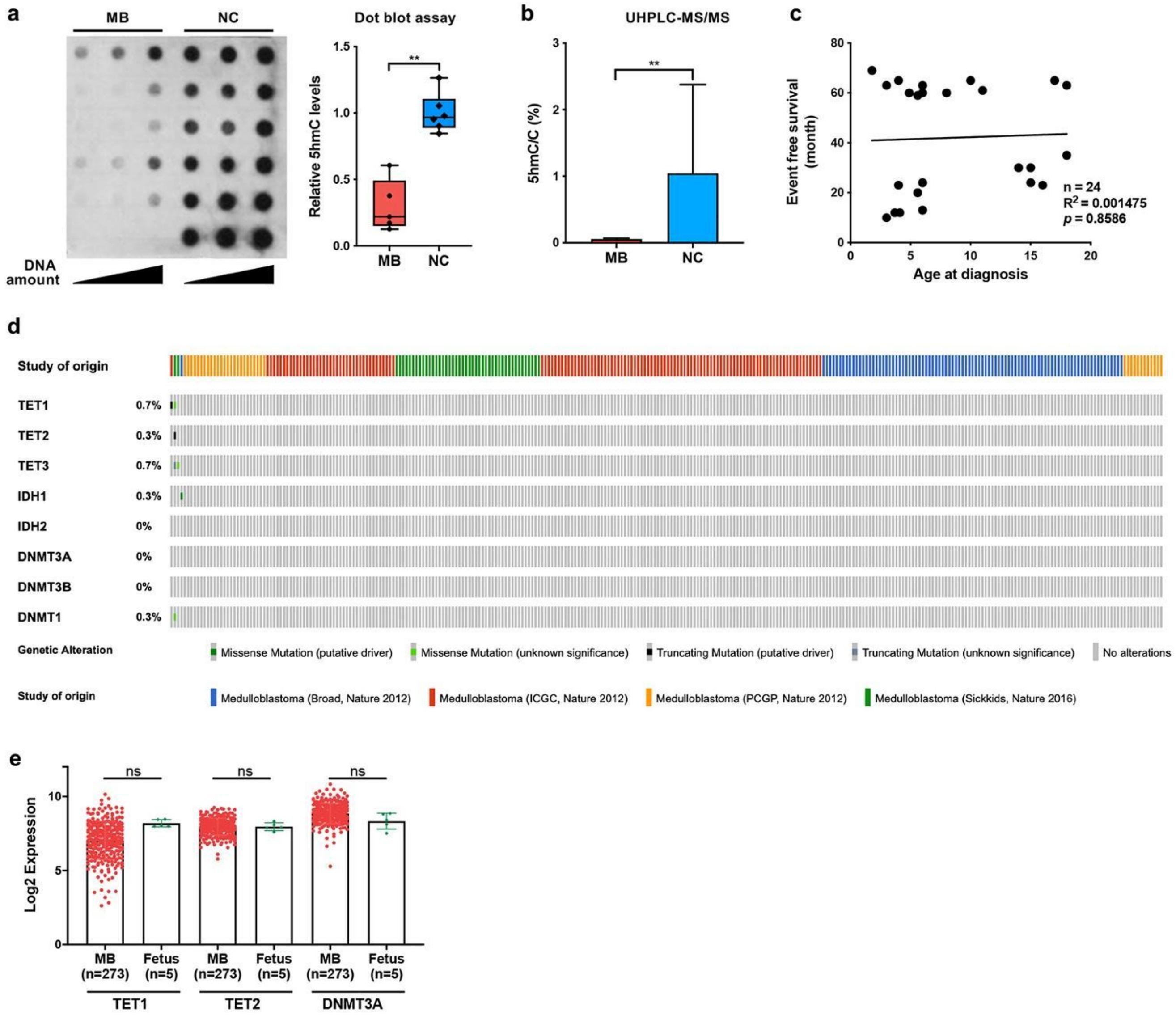

Fig S1. Loss of 5-hydroxymethylation is a hallmark of MBs. (a) 5hmC dot blot analysis using a different cohort shows a consistent decrease of total 5hmC levels in MBs (n=5) compared with age-matched normal cerebella (NC) (n=6) (**$p < 0.01$). (b) UHPLC-MS/MS result confirms decreased level of 5hmC in MBs (**$p < 0.01$; Additional file 20: Table S15). (c) Correlation between age at diagnosis in MBs and prognosis. Unlike 5hmC levels, age at diagnosis does not show linear correlation (R^2=0.001475, p = 0.8586). (d) Mutation profiles show a few putative driving mutations in the genes of enzymes involved in cytosine dynamics. (e) Expression levels of *TET1, TET2, and DNMT3A* in MBs (n=273) are comparable to the levels in fetus (n=5). (n.s. = not significant)

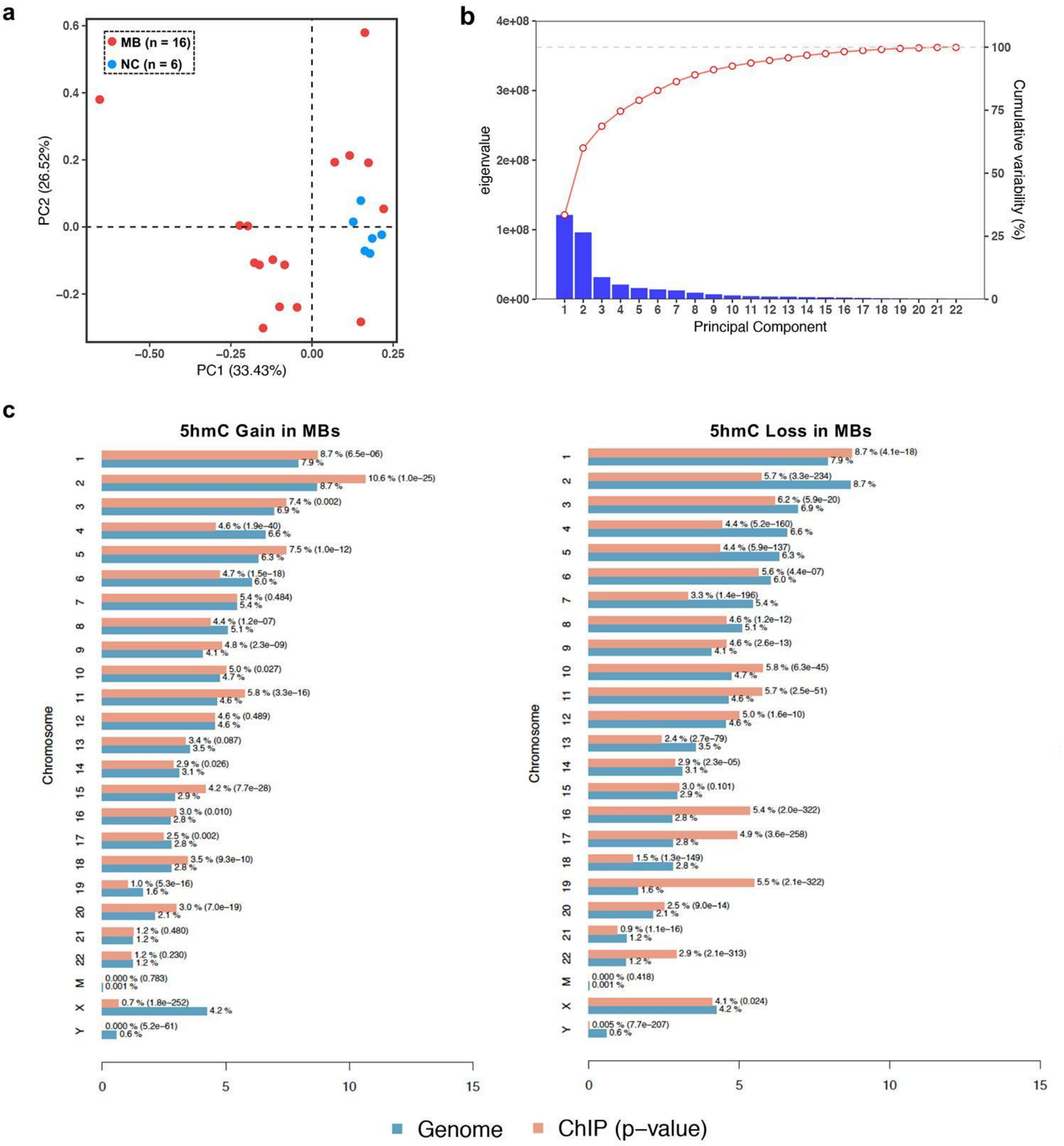

Fig S2. MB-associated DhMRs are implicated in stem-like properties. (a, b) Principal component analysis (PCA) scatter plot and scree plot of MB (n=16) and NC (n=6) samples using hMe-seal sequencing results. (c) Genomic annotation of 5hmC gain in MBs and 5hmC loss in MBs using CEAS.

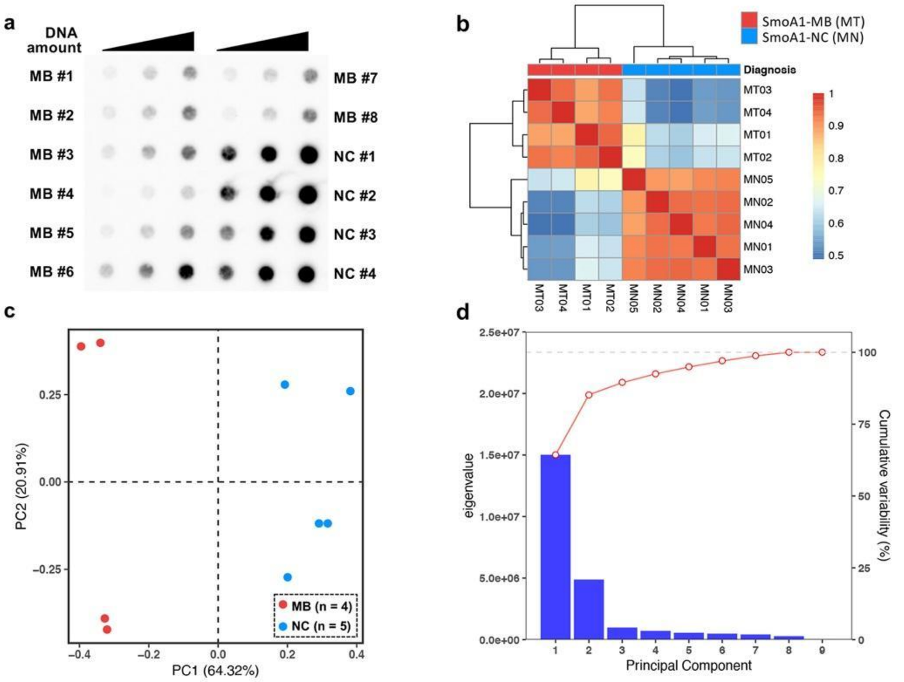

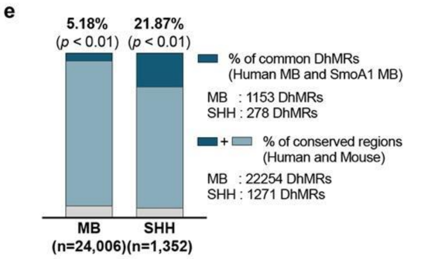

f

	Human MBs	SmoA1-MBs
No 5hmC regions	21,101	1,340,402
Gain-of-5hmC regions	1,153	22,368
Total regions	22,254	1,362,770

	SHH-MBs	SmoA1-MBs
No 5hmC regions	993	1,340,402
Gain-of-5hmC regions	279	22,368
Total regions	1,271	1,362,770

g

	Human MBs	SmoA1-MBs
No common genes	1,191	11,159
Common genes	3,514	9,051
Total genes	4,705	20,210

	SHH-MBs	SmoA1-MBs
No common genes	318	11,159
Common genes	461	9,051
Total genes	779	20,210

h

Genomic level	Human MBs
Loss-of-5hmC regions specific to human MBs	23,481
Commonly identified loss-of-5hmC regions	27,784 (54.20%)
Total regions	51,265

Gene level	Human MBs
No common genes	1,586
Commonly identified genes	8,599 (84.43%)
Total genes	10,185

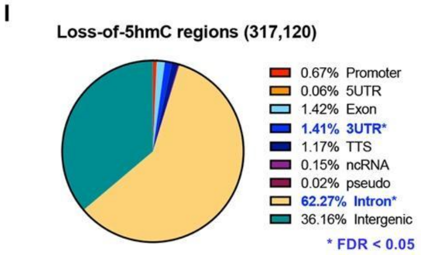

Fig S3. 5hmC signature in the SmoA1 mouse model recapitulates the human MB signature. (a) 5hmC dot blot analysis using SmoA1-MBs (n=8) and adjacent NCs (n=4). (b) Heatmap showing correlation of genomic 5hmC distribution among SmoA1-MB and NC samples. (c, d) Principal component analysis (PCA) scatter plot and scree plot of SmoA1-MB (n=4) and NC (n=5) samples using

hMe-seal sequencing results. (e) Bar graphs displaying commonly identified peaks in both human MBs (either 16 MBs or 4 SHH-MBs) and SmoA1-MBs. Grey indicates peaks identified from human MBs (n=1,752) and SHH-MBs (n=81) only but not in mouse, light green indicates peaks conserved in the mouse genome (human MBs n=21,101, SHH-MBs n=993) that were not identified in mouse MBs, and dark green indicates peaks which are commonly identified in both human and mouse MBs (human MBs n=1,153, SHH-MBs n=278). Peaks from both human MBs and SHH-MBs are significantly identified in SmoA1-MBs and the percentage are indicated above each bar ($p < 0.01$). For common peak identification, human 5hmC gain peaks (hg19) were converted to mouse peaks (mm10) using batch coordinate conversion (liftOver), and then common peaks were identified using intersectBed (bedtools). (f) Regional similarities between human MBs/SHH-MBs and SmoA1-MBs for the gain-of-5hmC regions. (g) Genic similarities between human MBs/SHH-MBs and SmoA1-MBs for the gain-of-5hmC regions. (h) Regional and Genic similarities between human MBs/SHH-MBs and SmoA1-MBs for the loss-of-5hmC regions. (i) Pie charts illustrating annotation summary of 5hmC loss in SmoA1-MB (n=317,120) using HOMER. Annotations with FDR < 0.001 compared to background are indicated in blue.

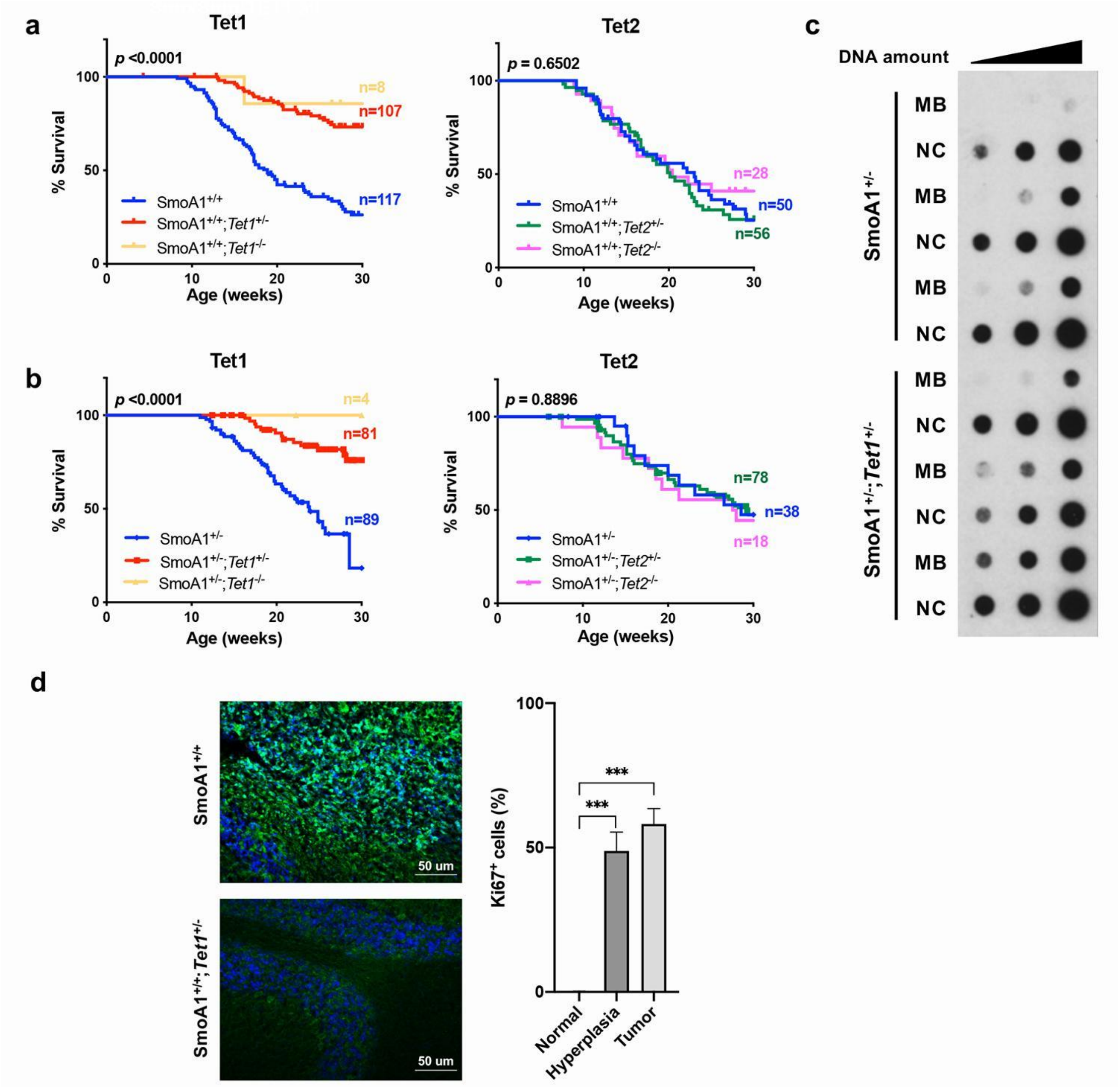

Fig S4. Elevated Tet1 is essential for MB progression. (a) Kaplan-Meier curves show the significant survival difference of only SmoA1$^{+/+}$ mice crossed with *Tet1* knock-out mice (Left: $p < 0.0001$; log rank test), but not crossed with *Tet2* knock-out mice ($p = 0.6502$; log rank test). (b)This phenomenon is consistently identified in SmoA1$^{+/-}$ crossed mice. (c) Consistent with SmoA1$^{+/+}$ mice, a significant increase in 5hmC levels was identified in SmoA1$^{+/-}$;*Tet1*$^{+/-}$ MBs (n=3) compared to SmoA1$^{+/-}$ MBs (n=3). (d) Representative immunofluorescence staining images of the cerebella obtained from either SmoA1$^{+/-}$ mice or SmoA1$^{+/-}$;*Tet1*$^{+/-}$ mice. There is no expression of Ki67 in normal cerebellar adjacent to MB, but hyperplasia and tumor show high levels of Ki67 in green (blue: DAPI; *** $p < 0.001$).

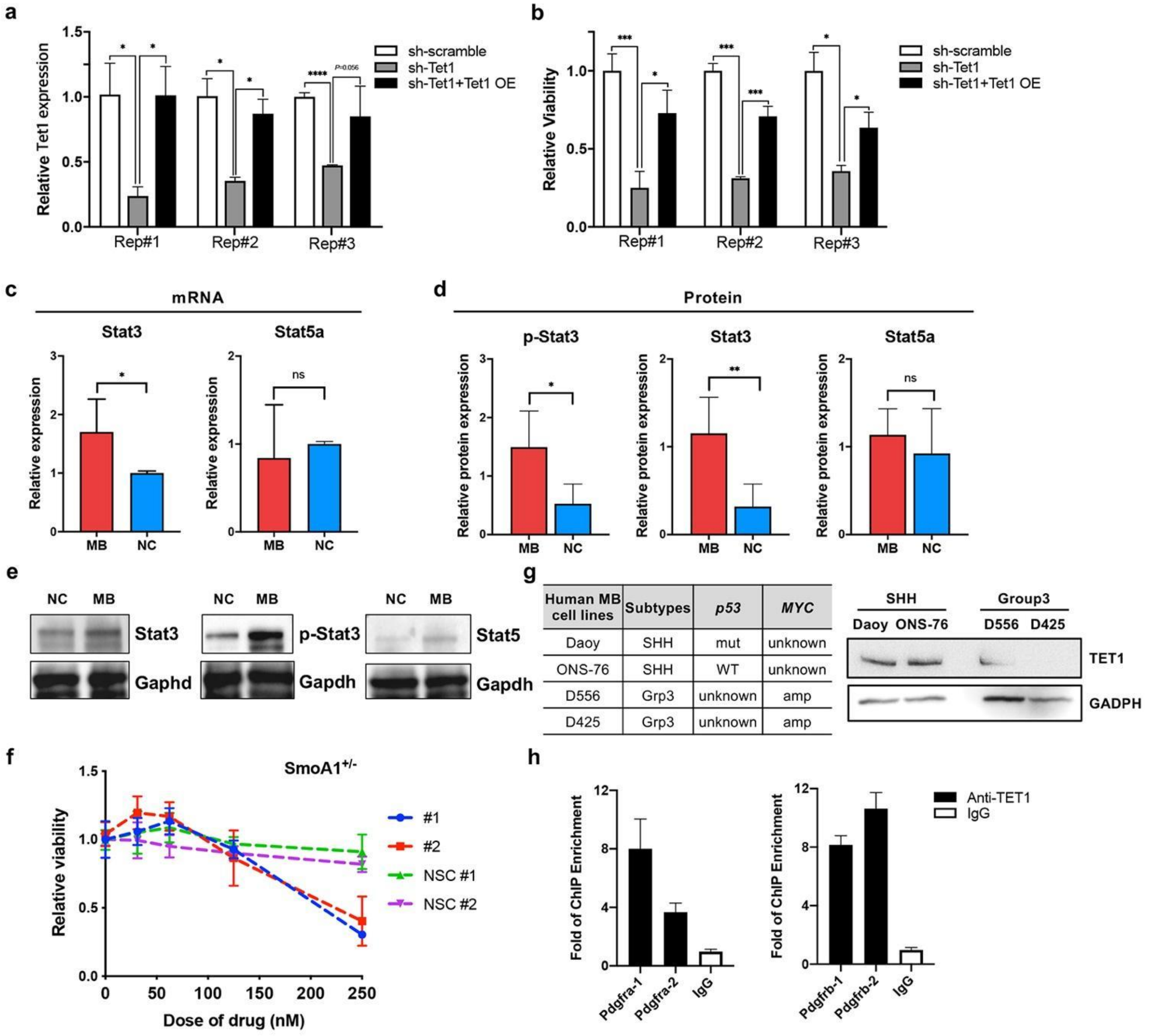

Fig S5. TET1 inhibition confers cytotoxic effect on both SmoA1- and human MBs. (a) Relative Tet1 expression of sh-Tet1-treated only or shTet1-treated with Tet1 overexpressed plasmid primary cells compared to sh-scrambled treated primary cells for three biological replicates. (b) Relative cell viability of sh-Tet1-treated only or shTet1-treated with Tet1 overexpressed plasmid primary cells compared to sh-scrambled treated primary cells for three biological replicates. False discovery rate (FDR) was determined with multiple comparison using one-way ANOVA test. * $p < 0.05$ and *** $p < 0.001$). (c) Only Stat3 mRNA level is overexpressed in SmoA1-MBs (* $p < 0.05$) compared to NCs. (d, e) Consistent with mRNA level, Stat3 protein level is overexpressed in SmoA1-MBs (** $p < 0.01$). In addition, p-Stat3 level is higher in SmoA1-MBs (* $p < 0.05$). (f) Relative cell viability depending on dose of drug (nM) in SmoA1$^{+/-}$. NSC: Neuronal stem cell. (g) Table showing characteristics of human MB cell line used in this study (left) and TET1 expression in each cell line (right). (h) TET1 is enriched at the regulatory

regions of Pdgfra and Pdgfrb in SmoA1-MBs as indicated in Figure 6e. Chromatin immunoprecipitation using anti-TET1 antibody was performed followed by qPCR.

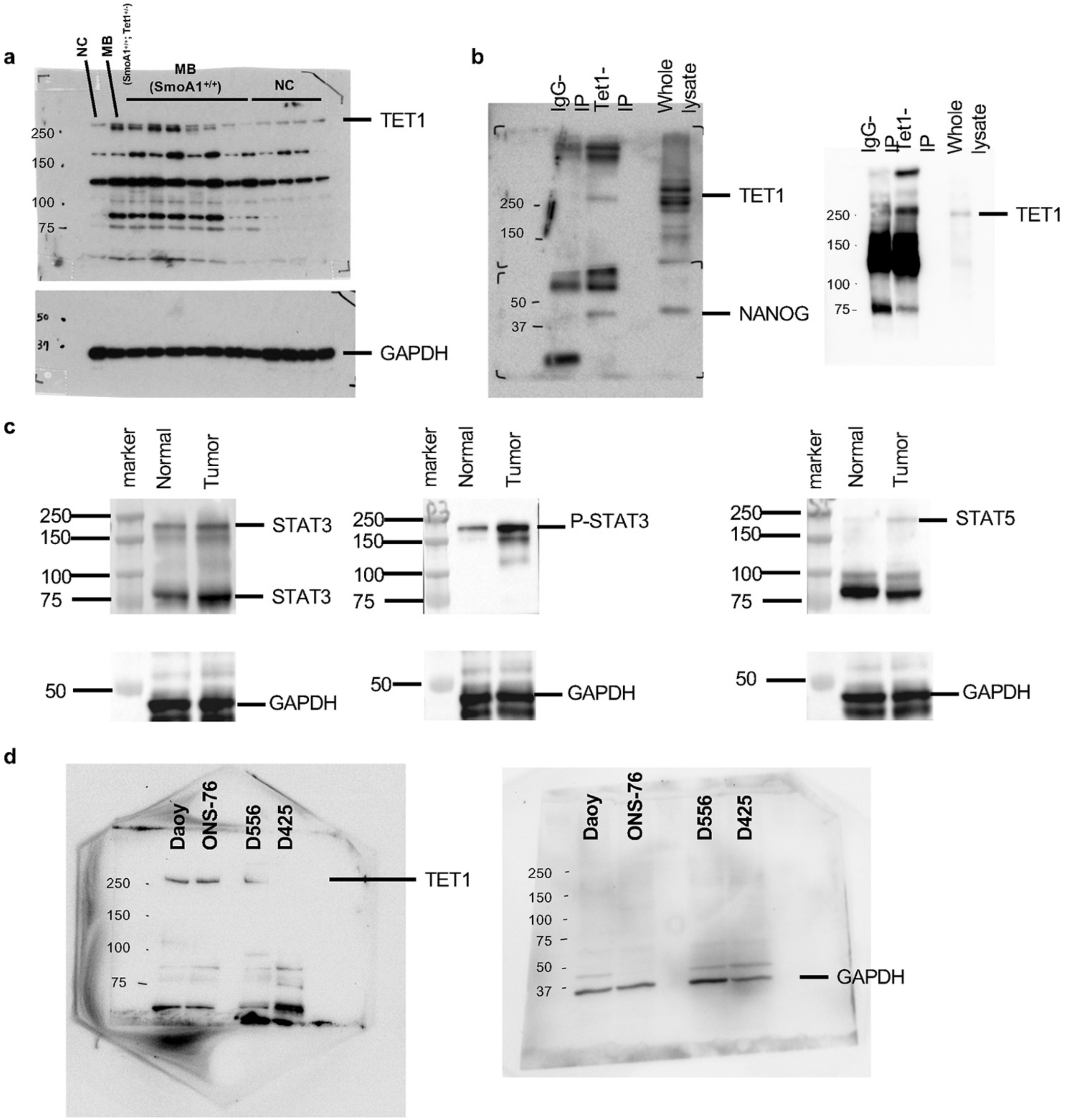

Fig S6. All full western blots in this study. (a) Raw western blots (Tet1 and Gapdh) for Fig. 4e. (b) Raw western blots (Tet1 and Nanog) for Fig. 6a. Tet1 was detected with two different antibodies: GTX124207 (left) and GTX627420 (right). (c) Raw western blots (Stat3, p-Stat3, Stat5) for Fig. S5e. The blots were cut and probed with different antibodies after transfer. (d) Raw western blots (TET1 and GAPDH) for Fig. S5g.
