# Supplementary material for: Ten-eleven translocation protein 1 modulates medulloblastoma progression
Source: Genome Biol. 2021 Apr 29;22:125. doi: 10.1186/s13059-021-02352-9 (PMC8082834; doi:10.1186/s13059-021-02352-9)
Supplement: Supplementary file 18 — Additional file 18. Review history. [file 13059_2021_2352_MOESM18_ESM.docx]

Review History

**First round of review**

**Reviewer 1**

**Are you able to assess all statistics in the manuscript, including the appropriateness of statistical tests used?** Yes, and I have assessed the statistics in my report.

**Comments to author:**

DNA modifications play a critical role in cancer biology.

Kim and co-authors investigate the alterations of 5hmC and TET enzymes in MB and their significance to cerebellar cancer formation. Their data have shown that total abundance of 5hmC is depleted in MB, but significantly enriched at regulatory regions of genes implicated in

stem-like properties and Nanog-binding motifs. The knockout of Tet1 in the SmoA1 mouse model attenuates uncontrolled proliferation, leading to a favorable prognosis. The pharmacological Tet1 inhibition reduces cell viability and PDGFsignaling pathway-associated genes.

The whole experiment is well designed, and the results are significant. This work will be broad interest to the pediatric cancer field.

However, the authors need to clarify or answer the following questions from the reviewer:

1. Although TETs control the 5hmC/5fC/5CaC, TETS still cross-talk to DNMTs in this pathway. The authors show that DNMT3A upregulated in MB. So, the authors may need to check whether or not DNMT3A also contribute to 5hmC regulation in MB? At least, DNMT3A might serve as a Tet1 blocker on the chromatin.

2. The DhMR is the key result in the paper, but authors only use DESeq2 to call the DhMR. The authors need to try one or two other methods, such as EdgeR model and SICER, to check the results will be similar or not.

3. The authors use RNAi to knockdown TET1, but did not show the TET1 rescue or over-expression experimental result here. Can Tet1 expression rescue the phenotype?

**Reviewer 2**

**Are you able to assess all statistics in the manuscript, including the appropriateness of statistical tests used?** Yes, and I have assessed the statistics in my report.

**Comments to author:**

In multiple kinds of tumors, a significant decrease of DNA 5hmC level is frequently observed and serves as a potentially robust indicator of poor survival. So far, there is no report yet as to how DNA 5hmC and TET enzymes are involved in medulloblastoma. In this manuscript, Kim H. et al reported their discovery of gain-of-5hmC region in medulloblastoma samples in contrast to the global depletion of 5hmC. They identified that the gain-of-5hmC regions were enriched at regulatory regions of genes implicated in stem-like properties and Nanog-binding motifs. Moreover, they found that Tet1 depletion in the SmoA1 MB model showed a more favorable prognosis and TET1 inhibition reduced cells viability. Overall, this is the first report to describe the DNA 5hmC profiles in medulloblastoma and the function of TET1 in medulloblastoma. However, the results shown in this manuscript are not well organized and lack compelling logic thus difficult to convince people of the conclusions that the authors claimed.

My Major concern is:

1. The interpretation of the results is not convincing enough:

a)	The authors declared that substantial enrichment (48.84 and 46.16% in the human and murine MBs, respectively) of gain-of-5hmC regions are identified at the Nanog binding sites. but actually, judged from their Table S5 and S11, up to 46.63% and 44.28% of background sequence contained the same motif. Meanwhile, if ranked by p value, Nanog-binding-site is not the most enriched one among all the motifs, therefore the conclusion that" gain-of-5hmC region is substantially enriched in the Nanog binding sites" is not convincing.

b)	Control samples are missing in several experiments. For example, loading control should be included when showing the results of dotblot assay to make sure that equal amount of DNA samples was loaded as the area of the dots varied a lot from one to another in Fig. 1a, 3a and 4c. In Fig. S5D, the band intensity of GAPDH, applied as loading control, are inconsistent among the four samples too.

c)	To investigate whether DhMRs in MBs are involved in controlling stem-like properties, the authors compared the gain-of-5hmC regions detected in human MB to those in the mouse fetal cerebellum and concluded that "5hmC patterns in human MBs are reminiscent of those found in fetal cerebellum". However, with R2= 0.00893, it is difficult to say "there is high correlation" in-between. In addition, in Figure 2f, it is written as R2= 0.0893, so which one is correct?

d)	According to Fig. 4c, SmoA1+/+, TET1+/- mice also had incidence of MB, which is conflicted with the results in Fig.4g that showed a 100% "normal" phenotype in the SmoA1+/+, TET1+/- mice.

e)	In Fig.6, the authors took advantage of meta-analysis to explain the role of TET1 in cooperation with Nanog in driving medulloblastoma tumorigenesis. However, the data they cited were originally obtained by using ESCs. Since the authors emphasized the difference between medulloblastoma and normal cerebellum in Fig.4, or between NSCs and primary medulloblastoma cells in Fig.5d, experimental evidence by using medulloblastoma-derived cells are necessary to verify their working models shown in Fig. 6g.

f)	With all the results presented in this manuscript, it is still unclear whether the role of TET1 in medulloblastoma is 5hmC-dependent due to the absence of experimental evidence. Does TET1 protein bind to the regulatory region of Pdgfra/b in 5hmC-dependent manner? Is the TET1 inhibition by UC-514321 dependent on 5hmC?

g)	There is no evidence in the manuscript to show that PI3k/akt signaling is activated by TET1, either directly or indirectly, as shown in their working model (Fig. 6g).

2. The quality of the figures needs to be improved.

a)	The HE analysis (Fig. 4g) and immunofluorescence analysis (Fig.4d) of mouse cerebellum should be repeated. In Fig.4g, the image quality is too low to be recognized, and scale bar should be included; In addition, since the authors calculated the ratio of hyperplasia and tumor occurrence, representative images to show such kind of phenotypes should be included in the manuscript. As to Fig.4d, what is shown in the image is just background or noise signal, but not Ki67- or Tet1-specific staining. It is the same for Fig. S4e.

b)	The resolution of the main figures is too low, especially for Fig. 2e, 3h, 4g, 4h, which are difficult to be recognized.

c)	When the authors described the results in the supplementary figures, the numbers of subpanel should be specified in the context in the same way as main figures.

d)	I could not tell the difference between Fig.4c and Fig. S3c, they appear to be the same but in different layout.

3. Questions for the authors:

a)	The authors identified 22,254 5hmC regions in human MBs, but 1,362,770 in mouse MB model, could the authors explain why there is such a huge difference between the two species?

b)	In Fig. S2a, the authors showed "a divergent 5hmC pattern", what is the possible reason? It is well established that medulloblastoma consists of 4 kinds of molecular subtypes and the molecular mechanism of their tumorigenesis vary from each other as evidenced from genomic, transcriptomic and DNA methylation analysis. Since all the four subtypes of MB are included in this study, it will be interesting to know whether the 5hmC profiles are different as well among the four subtypes. In addition, age should be taken into consideration as infant, child and adult medulloblastoma are distinct in their molecular mechanism as well.

c)	In Fig.S4a, only 8 TET1-/- mice were included for survival analysis which is not comparable to SmoA1+/+, and SmoA1+/+Tet1+/- mice (>100 for each). In addition, why the TET1+/- mice, instead of TET1-/- mice, were mainly focused in this study?

4. Suggestions for the authors:

a)	It has been widely accepted that LC-MS/MS is much more accurate and quantitative compared to dotblot analysis. Therefore, I would suggest to use LC-MS/MS analysis, rather than dotblot analysis, to compare the global 5hmC level among samples. The author performed this analysis only in Fig. S1B but not otherwhere.

b)	Since human tumor samples were used in this study, a statement to declare about the ethics approval and consent to participate should be included.

c)	Both TET1 and TET2 exhibited a higher RNA expression in MBs, but only TET1 knockout led to a better survival. Since the intention of this study is to characterize the role of 5hmC in MB, it will be interesting to examine the role of TET2 in that as the authors conclude that TET1 is not the major enzyme to establish global 5hmC level.

d)	Several independent studies have depicted the proteome landscape of medulloblastoma and found the deviation of the levels between mRNA and protein expression. Since it is the TET protein that catalyze the hydroxymethylation reaction, it is necessary to compare the TET expression at protein level to find out the key enzyme responsible for 5hmC change.

e)	The authors concluded that the cerebellum of SmoA1+/+, Tet1+/- is smaller than SmoA1+/+ based on the results of Fig. S4D. I did not find the detailed introduction how they compared the size of cerebella. In general, cerebella weight, HE analysis or MRI analysis is used for making an objective judgement, but not by gross observation only or by the area depicted by the dotted lines as shown in Fig. S4D. In addition, wild-type and age-matched mice should be included as a control.

f)	To show the change in protein expression or phosphorylation for Stat3 and Stat5, the original western blot analysis should be included, but not statistical analysis only.

**Authors Response**

**Point-by-point responses to the reviewers’ comments:**

**Reviewer #1:**

1. *“Although TETs control the 5hmC/5fC/5CaC, TETS still cross-talk to DNMTs in this pathway. The authors show that DNMT3A upregulated in MB. So, the authors may need to check whether or not DNMT3A also contribute to 5hmC regulation in MB? At least, DNMT3A might serve as a Tet1 blocker on the chromatin.”*

This is an excellent question. 5mC is a prerequisite to generate 5hmC and further cytosine modifications; in addition, DNMT3s are critical for de novo 5mC generation so that DNMT3s are overexpressed in various tumor types, leading to alteration of cytosine modifications. Additionally, the overexpression of DNMT3A is associated with poor prognosis to acute myeloid leukemia (AML) patients, suggesting its pivotal role in tumorigenesis. Although we identified a similar upregulation of DNMT3A in MB as with the other tumor types, there is a report that DNMT3A expression is not correlated with patients’ survival^1^. Based on this reviewer’s suggestion, we performed DNMT3A ChIP-seq using mouse MB cells and overlapped with the DhMRs that we detected. There was no significant overlap between DNMT3A binding and DhMRs. We are currently investigating whether the increased expression of DNMT3A could contribute to MB, but those analyses are beyond the scope of this manuscript.

^1^Timea Pocza et al., High expression of DNA methyltransferases in primary human medulloblastoma. *Folia Neuropathol* 54 (2): 105-113. (2016)

2. *“The DhMR is the key result in the paper, but authors only use DESeq2 to call the DhMR. The authors need to try one or two other methods, such as EdgeR model and SICER, to check the results will be similar or not.”*

To determine whether DhMRs called by DESeq2 were recurrently identified in different methods, we used edgeR^2^ to call the DhMRs with the same parameters used for DESe2. FDR < 0.01 and log2 fold change > 2 were considered as statistically significant. We identified 22,547 DhMRs that show increased hydroxymethylation in MBs (gain-of-5hmC) and 91,241 DhMRs that show decreased hydroxymethylation in MBs (loss-of-5hmC). Among 113,788 DhMRs, 18,099 regions were commonly identified as gain-of-5hmC in both DESeq2 and edgeR, while 63,627 regions were commonly identified as loss-of-5hmC. Therefore, approximately 75% of DhMRs identified using DESeq2 were also identified by the second method.

^2^Robinson MD, McCarthy DJ, Smyth GK (2010). “edgeR: a Bioconductor package for differential expression analysis of digital gene expression data.” Bioinformatics, **26**(1), 139-140. doi: [10.1093/bioinformatics/btp616](https://doi.org/10.1093/bioinformatics/btp616).

3. *“The authors use RNAi to knockdown TET1, but did not show the TET1 rescue or over-expression experimental result here. Can Tet1 expression rescue the phenotype?”*

This is a good suggestion. We have determined whether Tet1 overexpression could rescue the phenotype shown in knockdown Tet1. While the knockdown of Tet1 in primary cultures of SmoA1-MBs showed 69.43 % reduced viability compared to sh-scrambled treated primary cells, the overexpression of Tet1 in sh-Tet1 treated primary SmoA1 tumor cells showed a significant increase of viability (226.20%) compared to primary SmoA1 tumor cells treated with sh-Tet1 only, suggesting that Tet1 is critical for MB tumorigenesis.

**Reviewer #2:**

1. *“The authors declared that substantial enrichment (48.84 and 46.16% in the human and murine MBs, respectively) of gain-of-5hmC regions are identified at the Nanog binding sites. but actually, judged from their Table S5 and S11, up to 46.63% and 44.28% of background sequence contained the same motif. Meanwhile, if ranked by p value, Nanog-binding-site is not the most enriched one among all the motifs, therefore the conclusion that" gain-of-5hmC region is substantially enriched in the Nanog binding sites" is not convincing.”*

To conduct motif analysis with Homer, we performed CpG (%) normalization instead of GC%(G+C) normalization since cytokine modification is preferentially identified in CpG regions. In this case, we found the same percentage of Nanog sequence (48.84% and 46.16% in the human and murine MBs, respectively) as the percentage when running with default setting, but the same sequence was found in lower percentage of background sequence (45.64% and 44.08% in the human and murine MBs, respectively). The highly ranked motifs found in the human MBs, including Isl1 and Lhx3 (making Isl1-Lhx3 complex), are mostly implicated in motor neuron identity and neuronal development, but the Nanog motif is also ranked ninth. Considering that medulloblastomas contain normal neuronal features as well as oncogenic properties and that 5hmC plays a key role in cerebellar development, high enrichment of 5hmC at binding sites of TFs involved in both normal cerebellar development and tumorigenesis is makes more sense. Rank of the Nanog motif in the human MBs, including all molecular subtypes, is much higher than the rank in the mouse MBs (ranked 48^th^), indicating that 5hmC is enriched at the Nanog motif more commonly, regardless of molecular subtypes.

2. *“Control samples are missing in several experiments. For example, loading control should be included when showing the results of dot blot assay to make sure that equal amount of DNA samples was loaded as the area of the dots varied a lot from one to another in Fig. 1a, 3a and 4c.”*

We did perform methylene blue staining after every dot blot assay to make sure the samples were loaded equally (Fig. 1a, 3a, and 4c). In addition, we have now performed additional LC-MS/MS analyses to confirm our dot blot analyses.

3. *“To investigate whether DhMRs in MBs are involved in controlling stem-like properties, the authors compared the gain-of-5hmC regions detected in human MB to those in the mouse fetal cerebellum and concluded that "5hmC patterns in human MBs are reminiscent of those found in fetal cerebellum". However, with R2= 0.00893, it is difficult to say "there is high correlation" in-between. In addition, in Figure 2f, it is written as R2= 0.0893, so which one is correct?”*

Although the 5hmC signature in human MBs contains 5hmC regions found in both adult and fetus, gain of 5hmC regions are more highly enriched at 5hmC regions found in fetus compared to adult. Therefore, when we applied a regression model, we could get a significant p-value (p < 2.2 e-16). In addition, regarding R2, 0.0893 is correct.

4. *“According to Fig. 4c, SmoA1+/+, TET1+/- mice also had incidence of MB, which is conflicted with the results in Fig.4g that showed a 100% "normal" phenotype in the SmoA1+/+, TET1+/- mice.”*

At the time of the histological analysis the mice were 12 weeks old. In Fig. 4a, we did not observe any tumor incidence of SmoA1+/+, Tet1+/- mice at 12 weeks, showing that SmoA1+/+, Tet1+/- mice may develop the tumor or hyperplasia after 12 weeks old.

5. *“In Fig.6, the authors took advantage of meta-analysis to explain the role of TET1 in cooperation with Nanog in driving medulloblastoma tumorigenesis. However, the data they cited were originally obtained by using ESCs. Since the authors emphasized the difference between medulloblastoma and normal cerebellum in Fig.4, or between NSCs and primary medulloblastoma cells in Fig.5d, experimental evidence by using medulloblastoma-derived cells are necessary to verify their working models shown in Fig. 6g.”*

This is an excellent suggestion and we have attempted to perform TET1 ChIP-seq experiment using medulloblastoma cells. We have tried multiple TET1 ChIP experiments (over 10 times, with three different TET1 antibodies) followed by high-throughput sequencing, but unfortunately we could not get good quality data. The sequencing background was very high. However, TET1 ChIP DNA could be used for qPCR to examine specific loci. We did confirm the binding of Tet1 to the promoter regions of Pdgfra/b by qPCR (see the discussion below).

6. *“With all the results presented in this manuscript, it is still unclear whether the role of TET1 in medulloblastoma is 5hmC-dependent due to the absence of experimental evidence. Does TET1 protein bind to the regulatory region of Pdgfra/b in 5hmC-dependent manner?”*

As mentioned above, we performed TET1 ChIP-qPCR and found that TET1 could bind to the regulatory regions of Pdgfra/b marked by 5hmC.

7. *“There is no evidence in the manuscript to show that PI3k/akt signaling is activated by TET1, either directly or indirectly, as shown in their working model (Fig. 6g).”*

We agree with this comment. Although there are the reports that TET1 overexpression is linked to PI3K/AKT signaling, we did not see the direct connection between TET1 expression and PI3K/AKT signaling. We have removed the PI3K/AKT signaling in the figure and discussed the possible connection in the Discussion section.

8. *“The HE analysis (Fig. 4g) and immunofluorescence analysis (Fig.4d) of mouse cerebellum should be repeated. In Fig.4g, the image quality is too low to be recognized, and scale bar should be included; In addition, since the authors calculated the ratio of hyperplasia and tumor occurrence, representative images to show such kind of phenotypes should be included in the manuscript. As to Fig.4d, what is shown in the image is just background or noise signal, but not Ki67- or Tet1-specific staining. It is the same for Fig. S4e.”*

These are good suggestions and we have revised the figures accordingly. To understand specific staining of Tet1, we used Tet1 KO as a control. We clearly see that the Tet1 antibody used in this manuscript is specific to Tet1 and clear Tet1 expression in SmoA1-MBs, but not in Tet1-KO (Fig. 3).

**Fig. Expression of Tet1 in Tet1 KO and SmoA1-MB. Left: western blot image, right: Immuno-fluorescence image.**

9. *“The resolution of the main figures is too low, especially for Fig. 2e, 3h, 4g, 4h, which are difficult to be recognized.”*

We apologize for the inconvenience. In the revised manuscript, we have inserted the high-resolution images into the main text file.

10. *“When the authors described the results in the supplementary figures, the numbers of subpanel should be specified in the context in the same way as main figures.”*

We have corrected the numbers of subpanel according to the reviewer’s comment.

11. *“I could not tell the difference between Fig.4c and Fig. S3c, they appear to be the same but in different layout.”*

Fig. 4c used SmoA+/+ while Fig. S3c used SmoA+/-.

12. *“The authors identified 22,254 5hmC regions in human MBs, but 1,362,770 in mouse MB model, could the authors explain why there is such a huge difference between the two species?”*

We collected human medulloblastoma samples including four different subtypes which have divergent gene expression patterns while we used SmoA1 mouse model which recapitulates SHH medulloblastoma in human. In addition, genetic background of human samples is more complex than that of mouse samples, leading to substantially smaller number of 5hmC enrichment commonly identified by different individuals than mice.

13. *“In Fig. S2a, the authors showed "a divergent 5hmC pattern", what is the possible reason? It is well established that medulloblastoma consists of 4 kinds of molecular subtypes and the molecular mechanism of their tumorigenesis vary from each other as evidenced from genomic, transcriptomic and DNA methylation analysis. Since all the four subtypes of MB are included in this study, it will be interesting to know whether the 5hmC profiles are different as well among the four subtypes. In addition, age should be taken into consideration as infant, child and adult medulloblastoma are distinct in their molecular mechanism as well.”*

We tried to identify subtype-specific 5hmC signatures, but due to small sample size, differing ages and ethnicity, it was hard to determine whether the identified signature is unique to each subtype.

14. *“In Fig.S4a, only 8 TET1-/- mice were included for survival analysis which is not comparable to SmoA1+/+, and SmoA1+/+Tet1+/- mice (>100 for each). In addition, why the TET1+/- mice, instead of TET1-/- mice, were mainly focused in this study?”*

The easiest way to expand homozygote is to cross heterozygote with homozygote. However, because the homozygote of Tet1 is infertile, it is difficult to obtain enough mice by crossing heterozygote with heterozygote. Thus, we focused on the wild type and heterozygote mice and, fortunately, found that the heterozygote showed substantial survival improvement.

15. *“It has been widely accepted that LC-MS/MS is much more accurate and quantitative compared to dotblot analysis. Therefore, I would suggest to use LC-MS/MS analysis, rather than dotblot analysis, to compare the global 5hmC level among samples. The author performed this analysis only in Fig. S1B but not otherwhere.”*

This is a good suggestion. We have performed additional LC-MS/MS analyses to confirm our dot blot analyses.

16. *“Since human tumor samples were used in this study, a statement to declare about the ethics approval and consent to participate should be included.”*

We have included the following statement in the revised manuscript:

“All participants or their legal guardians enrolled in this study provided informed consent. All genetic analyses for this study were approved by the Institutional Review Board of Aflac cancer center, Emory University, and Xiangya Hospital.”

17. *“Both TET1 and TET2 exhibited a higher RNA expression in MBs, but only TET1 knockout led to a better survival. Since the intention of this study is to characterize the role of 5hmC in MB, it will be interesting to examine the role of TET2 in that as the authors conclude that TET1 is not the major enzyme to establish global 5hmC level.”*

This is an interesting suggestion. We have discussed this for future studies in the Discussion section.

18. “Several independent studies have depicted the proteome landscape of medulloblastoma and found the deviation of the levels between mRNA and protein expression. Since it is the TET protein that catalyze the hydroxymethylation reaction, it is necessary to compare the TET expression at protein level to find out the key enzyme responsible for 5hmC change.”

This is a good suggestion. Because protein level is a more direct method to prove overexpression, we used protein samples obtained from SmoA1 mouse model to understand Tet1 expression in the tumors. Unfortunately, the human tumor samples contained lots of blood and we did not have sufficient materials for protein analyses.

19. *“The authors concluded that the cerebellum of SmoA1+/+, Tet1+/- is smaller than SmoA1+/+ based on the results of Fig. S4D. I did not find the detailed introduction how they compared the size of cerebella. In general, cerebella weight, HE analysis or MRI analysis is used for making an objective judgement, but not by gross observation only or by the area depicted by the dotted lines as shown in Fig. S4D. In addition, wild-type and age-matched mice should be included as a control.”*

This is a good suggestion. Unfortunately, we did not collect cerebella weight information or perform MRI analysis, but we did HE analysis when we collected samples. It would be challenging to re-do this analysis at this stage.

20. *“To show the change in protein expression or phosphorylation for Stat3 and Stat5, the original western blot analysis should be included, but not statistical analysis only.”*

We have included the western blot with SmoA1-MB and matched normal to confirm whether Stat3, and p-Stat3 were truly overexpressed in tumor tissue while Stat5 was unchanged. As shown in the figure below, p-Stat3 was clearly overexpressed, and Stat3 and Stat5 were moderately overexpressed in tumor sample compared to normal sample.

**Fig. Expression of Stat3, p-Stat3, and Stat5 in SmoA1-MB and matched normal.** N indicates normal sample while T indicates tumor sample.

**Second round of review**

**Reviewer 1**

The authors designed more experiments to answer most of the reviewer's questions.

However, there are still several points need to be clarified:

1. The authors should explain the difference of the ratio in intron and intergenic regions in figure 2b and figure 3c. Is it because of the data itself or genomic background?

2. Tet1 staining of MB in Figure 4h is not clear. It is hard to tell whether Tet1 and Ki67 actually in the same cells. Also, there is no scale bar in this figure.

3. In the figure 5i, is PDGF the only pathway that is significantly enriched? If there are other pathways enriched, please show other pathways, so that reader can get the whole picture.

**Reviewer 2**

In the revised version, the authors answered all the questions raised by the reviewers. I appreciate their effort to address my concern; however, I think it is still premature to be accepted for publication in Genome Biology due to the following reasons:

1. There are two parts in this manuscript, with the first part focusing on 5hmC analysis and the 2nd part focusing on Tet1 in MB. Nevertheless, although the authors identified the change of 5hmC and Tet1 in MB, and found that Tet1 knockout could delay the tumor onset and progression, there is no direct or sufficient evidence showing that the role of Tet1 in MB is dependent on 5hmC. Particularly, in figure 6, it is unclear whether 5hmC is involved in the binding of Tet1 protein to Pdgfa/b and their gene expression control. Therefore, it is premature to draw a conclusion that “Tet1-mediated 5hmC alteration modulates medulloblastoma”. Overall, I feel that the line between robust experimentally validated conclusions and the author’s own speculation is sometimes blurred in this manuscript.

2. The results of several experiment are unconvincing due to insufficient evidence or low quality.

a)	Loading control is necessary to be included in the dot blot assay. For example, in Fig. 1a, the sizes of circles between NC and MB varied significantly, I would wonder whether equal amount of DNA was loaded without methylene blue staining as loading control. since the authors said that they have performed methylene blue staining, the results should be shown in the figures.

b)	The quality of HE analysis and immunofluorescence are not high enough to make a solid conclusion. For morphology analysis in the brain, only paraffin or frozen sections at the same positions could be chosen for comparison. however, this was not the case for all the IF or HE results and thereby they were not comparable. In addition, the quality of images in Fig.4h is too low to be convincing, the pattern of Ki67 and Tet1 staining are strange. In addition, how to explain that TET1 expression in NC is completely negative? and why TET1 was expressed in 10% of K67+ cells only in MB? this result is somewhat contradictory to the significant difference shown in Fig.4e.

c)	In the response letter, the application of TET1 in western blot analysis is convincing. however, the images of Tet1 immunofluorescence are not reliable. First, the author should perform IF by using the frozen sections at the same position for comparison; Second, could the author explain which brain region was shown in this image, as it is hard to understand why TET1 exhibits such kind of pattern in the brain.

d)	Due to the insufficient detail of the methods, it is unclear how the author defined the region of promoter. In Fig. 6. Pdgfa/Pdgfb were identified due to their DhMRs located at promoter-TTS regions. however, in Fig. 6e, the peak selected does not seem to be located in promoter region as the author stated in the response letter. It is unclear either that which regions in Pdgfa/b were amplified for CHIP-qPCR.

3. Their conclusion was not well supported by the data show in the manuscript.

a)	I suggest that the authors should be careful to avoid being subjective in analyzing the results. For example, only the significance of Nanog motif among all the gain-of-5hmC regions although it is not the most significant one; With a correlation coefficient as low as 0.0893, it was concluded that 5hmC patterns in human MB are reminiscent of those found in fetal cerebellum (Fig. 2); In Fig2e, the authors highlighted the notch signaling pathway, which is not the most enriched one either. Similarly, in Fig.5i, the authors concluded that the PDGF pathway is significantly enriched. Is the only pathway to be identified? otherwise, I think it is somewhat not objective to make such conclusion without showing other enriched pathways.

b)	The author stated that SmoA1+/+Tet1+/- mice had a delay in tumor onset, while the experimental evidence is missing in the manuscript.

c)	The result in figure 4d does not support their conclusion that SmoA1+/+ mice showed significantly larger cerebella without solid experiment data.

d)	In figure S4e, it is surprising that SmoA1+/+ mice was negative in Ki67 staining and normal cerebellar morphology was shown instead of tumor.

e)	I did not find the corresponding figure to support their conclusion in line 52-57, page 17.

f)	In line 21-26, page 19, the authors stated that “in MB samples, but not normal cerebella, Nanog and Tet1 bind to the regulatory region of Pdgfa/b”, I think it is overstatement to draw such a conclusion without experimental evidence to confirm it, IGV data from sequencing data is not suffficient.

g)	In contrast to 1362779 5hmC regions detected in mouse MB model, only 22254 5hmC regions in human MBs were analyzed in this study. I am afraid that a lot of important information is missing as there should exist much greater number of 5hmC regions in human MB as well.

4. The approaches used in this work are not clearly explained and incomplete. For example, I could not find the method of CHIP-qPCR, LC-MS/MS analysis. In the MB mouse model, could the author explain how they define hyperplasia from tumor and provide evidence for that? In addition, the file “key resource table” could not be found either. As to bioinformatic analysis, the author should describe in more detail how they compare the 5hmC region between human and mouse, which is rarely seen in literature. In addition, it is unclear how they performed enrichment analysis such as GREAT.

5. In figure 3, the authors made a comparison between human MB and mouse MB model. why the author only compared gain-of-5hmC region, but not loss-of-5hmC region?

6. Minor concern,

a)	I am curious that how about the role of TET3 in MB and why it is not included in this study.

b)	In figure 5a, why TET2 was affected when knocking down TET1 in primary MB cells?

c)	“MB, NC”, “N, T”, it seems that different names were used for the same kind of samples throughout the context, which is quite confusing to understand.

d)	Several mistakes in figures layout or description of their results.

**Authors Response**

**Point-by-point responses to the reviewers’ comments:**

**Reviewer #1:**

**1.** *“The authors should explain the difference of the ratio in intron and intergenic regions in figure 2b and figure 3c. Is it because of the data itself or genomic background?”*

Because human and mouse have different sizes of genome, intron and intergenic regions, the ratio in Figure 2b (human) is different from that in Figure 3f/3c (mouse).

**2.** *“Tet1 staining of MB in Figure 4h is not clear. It is hard to tell whether Tet1 and Ki67 actually in the same cells. Also, there is no scale bar in this figure.”*

We have provided a new panel with scale bar in the revised manuscript.

**3.** *“In the figure 5i, is PDGF the only pathway that is significantly enriched? If there are other pathways enriched, please show other pathways, so that reader can get the whole picture.”*

When treated with TET1 inhibitor, we identified 643 genes, including mitogen-activated protein kinases (*Mapk3*, *Mapk8*, and *Mapk7*) and *Pik3c*, significantly downregulated. Using these genes, we investigated which signaling pathway(s) was significantly enriched using PANTHER, and interestingly, we only found that PDGF signaling pathway was significantly enriched.

**Reviewer #2:**

**1.** *“There are two parts in this manuscript, with the first part focusing on 5hmC analysis and the 2nd part focusing on Tet1 in MB. Nevertheless, although the authors identified the change of 5hmC and Tet1 in MB, and found that Tet1 knockout could delay the tumor onset and progression, there is no direct or sufficient evidence showing that the role of Tet1 in MB is dependent on 5hmC. Particularly, in figure 6, it is unclear whether 5hmC is involved in the binding of Tet1 protein to Pdgfa/b and their gene expression control. Therefore, it is premature to draw a conclusion that “Tet1-mediated 5hmC alteration modulates medulloblastoma”. Overall, I feel that the line between robust experimentally validated conclusions and the author’s own speculation is sometimes blurred in this manuscript.”*

We understand this reviewer’s concern and agree that we do not have the sufficient data to demonstrate the role of Tet1 in MB is 5hmC-dependent. Therefore we have changed the title of this manuscript to “Ten-Eleven Translocation Protein 1 modulates medulloblastoma progression”, removed the model figure (Figure 6g), and toned down our overall conclusion.

**2.** *“The results of several experiment are unconvincing due to insufficient evidence or low quality.*

*a) Loading control is necessary to be included in the dot blot assay. For example, in Fig. 1a, the sizes of circles between NC and MB varied significantly, I would wonder whether equal amount of DNA was loaded without methylene blue staining as loading control. since the authors said that they have performed methylene blue staining, the results should be shown in the figures.*

This is a good point. Unfortunately, the data were produced almost five years ago, and the person who performed the initial analyses has since left the lab. We were only able to locate some methylene blue staining blots. We only have a limited amount of human MB DNA samples left in the lab, which would not be sufficient to repeat the 5hmC dot blot assay. So we decided to remove the dot blot data for Figure 1a and only present our UHPLC-MS/MS data in the revised manuscript. We have included all the UHPLC-MS/MS quantification data as Supplementary Table in the revised manuscript.

*b) The quality of HE analysis and immunofluorescence are not high enough to make a solid conclusion. For morphology analysis in the brain, only paraffin or frozen sections at the same positions could be chosen for comparison. however, this was not the case for all the IF or HE results and thereby they were not comparable. In addition, the quality of images in Fig.4h is too low to be convincing, the pattern of Ki67 and Tet1 staining are strange. In addition, how to explain that TET1 expression in NC is completely negative? and why TET1 was expressed in 10% of K67+ cells only in MB? this result is somewhat contradictory to the significant difference shown in Fig.4e.*

We have provided a new panel of images in Figure 4h to show differential expression of Ki67 and Tet1 expression in MB and adjacent normal cerebellum. Due to abnormality, hyperplasia, and tumor in cerebellum of SmoA1+/+, it is challenging to exactly match the same position, but we tried to take pictures at comparable position at the cerebellar posterior lobe, where tumors develop most. Although our HE staining images are not perfect, they are of sufficient quality to show morphological changes, which are similar to multiple previously published works (sample figures from previously published works are attached at the end of this response). The difference in 4e and 4h could be caused by the difference in the sensitivity of two methods (Western blot with whole ground tissue vs immunofluorescence in each individual cell) and heterogeneity of tumor cells, which could have detection limits in low expressing cells, and could also be affected by the sensitivity of a given antibody.

*c) In the response letter, the application of TET1 in western blot analysis is convincing. however, the images of Tet1 immunofluorescence are not reliable. First, the author should perform IF by using the frozen sections at the same position for comparison; Second, could the author explain which brain region was shown in this image, as it is hard to understand why TET1 exhibits such kind of pattern in the brain.*

To demonstrate TET1 overexpression is found in the tumor region but not in the normal region, we selected 12 week-old mice (6 weeks earlier than the median age of tumor-associated symptoms). At this age, we could identify some mice that have tumors which did not fully cover the cerebellum. Thus, we identified areas which contain both the abnormally proliferated regions and the normal regions to demonstrate that Tet1 was overexpressed in the abnormal regions.

We tested three Ki67 antibodies and seven Tet1 antibodies for immunofluorescence staining in the cerebellum and medulloblastoma. To show the specificity of the Tet1 antibody that we used, Tet1 knockout cerebellum is shown. SmoA1-medulloblastoma region with high Tet1 expression and MB adjacent normal cerebellum posterior lobule with little or less Tet1 is shown in the figure below.

**Figure. Expression of Tet1 protein. Left: western blot image, right: Immuno-fluorescence image.**

*d) Due to the insufficient detail of the methods, it is unclear how the author defined the region of promoter. In Fig. 6. Pdgfa/Pdgfb were identified due to their DhMRs located at promoter-TTS regions. however, in Fig. 6e, the peak selected does not seem to be located in promoter region as the author stated in the response letter. It is unclear either that which regions in Pdgfa/b were amplified for CHIP-qPCR.*

We defined the promoter region as the 1kb upstream of the transcription start site. ChIP-qPCR experiment was used to confirm ChIP-seq findings. We have now tested an additional site that is within the Pdgfa/Pdgfb promoter regions and included the data in the revised manuscript.

**3.** *“Their conclusion was not well supported by the data show in the manuscript.*

*a) I suggest that the authors should be careful to avoid being subjective in analyzing the results. For example, only the significance of Nanog motif among all the gain-of-5hmC regions although it is not the most significant one; With a correlation coefficient as low as 0.0893, it was concluded that 5hmC patterns in human MB are reminiscent of those found in fetal cerebellum (Fig. 2); In Fig2e, the authors highlighted the notch signaling pathway, which is not the most enriched one either.”*

We agree with this comment and have revised our manuscript accordingly.

*Similarly, in Fig.5i, the authors concluded that the PDGF pathway is significantly enriched. Is the only pathway to be identified? otherwise, I think it is somewhat not objective to make such conclusion without showing other enriched pathways.*

As indicated above, when treated with TET1 inhibitor, we identified 643 genes, including mitogen-activated protein kinases (*Mapk3*, *Mapk8*, and *Mapk7*) and *Pik3c*, significantly downregulated. Using these genes, we investigated which signaling pathway(s) was significantly enriched using PANTHER, and interestingly, we only found that PDGF signaling pathway was significantly enriched.

*b) The author stated that SmoA1+/+Tet1+/- mice had a delay in tumor onset, while the experimental evidence is missing in the manuscript.*

We apologize for this omission. Hunched posture is the distinguishing feature found in tumor-bearing mice. We performed Kaplan-Meier analysis using onset of symptoms with SmoA1^+/+^;Tet1^+/-^ and SmoA1^+/+^;Tet1^+/+^ mice, and concluded that SmoA1^+/+^;Tet1^+/-^ had a delay in tumor onset. We have included additional explanation in the revised manuscript.

*c) The result in figure 4d does not support their conclusion that SmoA1+/+ mice showed significantly larger cerebella without solid experiment data.*

We apologize for this mistake and have removed this statement in our revised manuscript.

*d)In figure S4e, it is surprising that SmoA1+/+ mice was negative in Ki67 staining and normal cerebellar morphology was shown instead of tumor.*

We apologize for this error. SmoA1+/+ was mislabeled and we have corrected labels in the supplementary figure.

*e) I did not find the corresponding figure to support their conclusion in line 52-57, page 17.*

We apologize for the confusion. We have revised this section and indicated the corresponding figures clearly.

*f)  In line 21-26, page 19, the authors stated that “in MB samples, but not normal cerebella, Nanog and Tet1 bind to the regulatory region of Pdgfa/b”, I think it is overstatement to draw such a conclusion without experimental evidence to confirm it, IGV data from sequencing data is not sufficient.*

We agree with this comment and have revised the text accordingly.

*g) In contrast to 1362779 5hmC regions detected in mouse MB model, only 22254 5hmC regions in human MBs were analyzed in this study. I am afraid that a lot of important information is missing as there should exist much greater number of 5hmC regions in human MB as well.*

This is a good question. In our analyses, we focused on the genomic regions with altered 5hmC consistently across multiple patients’ samples. Comparing to the SmoA1 mouse model that recapitulates SHH subtype and is genetically more homogenous, patients’ samples were collected from different genetic background and diverse molecular subtypes, making the patient samples highly heterogeneous. Given the intrinsic heterogeneity with patients’ samples, we detected fewer common 5hmC alterations across human samples.

**4.** The approaches used in this work are not clearly explained and incomplete. For example, I could not find the method of CHIP-qPCR, LC-MS/MS analysis. In the MB mouse model, could the author explain how they define hyperplasia from tumor and provide evidence for that? In addition, the file “key resource table” could not be found either. As to bioinformatic analysis, the author should describe in more detail how they compare the 5hmC region between human and mouse, which is rarely seen in literature. In addition, it is unclear how they performed enrichment analysis such as GREAT.

We apologize for the inconvenience. We have now included the method of ChIP-qPCR and UHPLC-MS/MS analysis in our updated manuscript. We also summarized all materials in “key resource table” (Supplementary Table S16).

Based on the original paper that established the SmoA1 mouse model, hyperplasia was defined as less than 50% of the cerebellum morphologically abnormal while tumor was defined as more than 50% of the cerebellum morphologically abnormal (Hallahan AR et al, *Cancer Res* 2004; Hatton BA et al, *Cancer Res* 2008). Accordingly, we observed abnormal proliferated regions at the external granular cell layer with distinct cerebellar folded structures in the hyperplasia samples, while bulk proliferated mass without folded structure was found in tumor samples. We have included this information in the revised manuscript.

Regarding bioinformatic analysis, we think this comparison is important to show that this epigenetic mark is conservative and tissue- and disease-specific. To do this analysis across the genome, we first converted human genomic regions into mouse genomic regions using the LiftOver tool, and then compared the human regions with 5hmC regions identified in the SmoA1 mouse model. At the gene level, we first annotated and then identified orthologs using BioMart. You can find the detailed description in “Analysis of hMe-seal sequencing data to identify DhMRs”. For enrichment analysis using GREAT, we used bed files of gain-of-5hmC or loss-of-5hmC using default “Basal plus extension” settings.

**5.** In figure 3, the authors made a comparison between human MB and mouse MB model. why the author only compared gain-of-5hmC region, but not loss-of-5hmC region?

This is a good suggestion. We compared loss-of-5hmC of human MB and mouse MB with the same procedure. Interestingly, we identified substantial overlap of loss-of-5hmC between human MB and mouse in the genomic level (54.20%) and gene level (84.43%). Although we hypothesize that gain-of-5hmC regions can play an active role in cancer progression and be more associated with subtype specific gene expression, loss-of-5hmC may also play a critical role in tumorigenesis. We have included these analyses in the revised manuscript.

- Genomic level

|  | **Human MBs** |
| --- | --- |
| **Loss-of-5hmC regions specific to human MBs** | 23,481 |
| **Commonly identified loss-of-5hmC regions** | 27,784 (54.20%) |
| **Total regions** | 51,265 |

- Gene level

|  | **Human MBs** |
| --- | --- |
| **No common genes** | 1,586 |
| **Commonly identified genes** | 8,599  (84.43%) |
| **Total genes** | 10,185 |

**6.** Minor concern,

a) I am curious that how about the role of TET3 in MB and why it is not included in this study.

This is a good question. Tet3 KO is embryonic lethal, suggesting Tet3 plays a critical role in embryonic development. As shown in Figure 1e, we did not find abnormal overexpression in medulloblastoma and that’s why we focused on Tet1 and Tet2 in this study.

b) In figure 5a, why TET2 was affected when knocking down TET1 in primary MB cells?

This is a good question. It is unclear why TET2 expression was affected since shRNA sequence is specific to TET1. It has been reported that Tet1 could interact with Tet2. It is possible that the absence of Tet1 could alter the expression of Tet2, which will require further investigation.

*c) “MB, NC”, “N, T”, it seems that different names were used for the same kind of samples throughout the context, which is quite confusing to understand.*

We apologize for the inconsistency and have corrected them.

*d) Several mistakes in figures layout or description of their results.*

Thanks for pointing them out and we have corrected the figure legend (Figure 4) based on our final figures.

**Cerebellum HE Staining from previous publications:**

1.Lateral cerebellum is preferentially sensitive to high sonic hedgehog signaling and medulloblastoma formation^1^
2.Generation of a Mouse Model of Atypical Teratoid/Rhabdoid Tumor of the Central Nervous System through Combined Deletion of Snf5 and p53^2^
3.Specific Targeting of Brain Tumors with an Optical/Magnetic Resonance Imaging Nanoprobe across the Blood-Brain Barrier^3^
4.Disrupting the CD47-SIRPα anti-phagocytic axis by a humanized anti-CD47 antibody is an efficacious treatment for malignant pediatric brain tumors^4^
5.mTORC1-Mediated Inhibition of 4EBP1 Is Essential for Hedgehog Signaling-Driven Translation and Medulloblastoma^5^
6.Aberrant ERBB4-SRC Signaling as a Hallmark of Group 4 Medulloblastoma Revealed by Integrative Phosphoproteomic Profiling^6^
7.Opposing Tumor-Promoting and -Suppressive Functions of Rictor/mTORC2 Signaling in Adult Glioma and Pediatric SHH Medulloblastoma^8^

References:

1.	Tan, I.-L., et al. Lateral cerebellum is preferentially sensitive to high sonic hedgehog signaling and medulloblastoma formation. Proceedings of the National Academy of Sciences 115, 3392-3397 (2018).

2.	Ng, J.M.Y., et al. Generation of a Mouse Model of Atypical Teratoid/Rhabdoid Tumor of the Central Nervous System through Combined Deletion of Snf5 and p53. Cancer Research 75, 4629-4639 (2015).

3.	Veiseh, O., et al. Specific Targeting of Brain Tumors with an Optical/Magnetic Resonance Imaging Nanoprobe across the Blood-Brain Barrier. Cancer Research 69, 6200-6207 (2009).

4.	Gholamin, S., et al. Disrupting the CD47-SIRPα anti-phagocytic axis by a humanized anti-CD47 antibody is an efficacious treatment for malignant pediatric brain tumors. Science Translational Medicine 9, eaaf2968 (2017).

5.	Wu, C., et al. mTORC1-Mediated Inhibition of 4EBP1 Is Essential for Hedgehog Signaling-Driven Translation and Medulloblastoma. Developmental Cell 43, 673-688.e675 (2017).

6.	Forget, A., et al. Aberrant ERBB4-SRC Signaling as a Hallmark of Group 4 Medulloblastoma Revealed by Integrative Phosphoproteomic Profiling. Cancer Cell 34, 379-395.e377 (2018).

7.	Akgül, S., et al. Opposing Tumor-Promoting and -Suppressive Functions of Rictor/mTORC2 Signaling in Adult Glioma and Pediatric SHH Medulloblastoma. Cell Reports 24, 463-478.e465 (2018).

**Third round of review**

**Reviewer 2**

In this revised manuscript, the authors have addressed most questions I raised before. Therefore, I recommend accepting it to be published in Genome Biology.
